# Use of a ferroptosis-related gene signature to construct diagnostic and prognostic models for assessing immune infiltration in metabolic dysfunction-associated fatty liver disease

**DOI:** 10.3389/fcell.2023.1199846

**Published:** 2023-10-19

**Authors:** Xin Lian, Xulei Tang

**Affiliations:** The First Clinical Medical College of Lanzhou University, Lanzhou, Gansu, China

**Keywords:** metabolic dysfunction-associated fatty liver disease, ferroptosis, immune infiltration, bioinformatics, differentially expressed genes, ferroptosis-related genes

## Abstract

**Introduction:** Metabolic dysfunction-associated fatty liver disease (MAFLD), a serious health problem worldwide, can involve ferroptosis. This study aimed to comprehensively analyze the ferroptosis-related genes associated with MAFLD.

**Methods**: Ferroptosis-related differentially expressed genes (FRDEGs) were identified in patients with MAFLD and healthy individuals. Gene ontology functional enrichment analysis, Kyoto Encyclopedia of Genes and Genomes pathway enrichment analysis, and gene set enrichment analysis (GSEA) were used to analyze the relevant action pathways of the FRDEGs. The Encyclopedia of RNA Interactomes, CHIPBase, and comparative toxicogenomics databases were used to build mRNA-miRNA, mRNA-transcription factor (TF), and mRNA-drug interaction networks, respectively. A diagnostic model was constructed and bioinformatics analysis methods, such as least absolute shrinkage and selection operator regression analysis, Cox regression analysis, nomogram-based analysis, consensus clustering analysis, and single-sample GSEA, were used to systematically investigate the prognostic values and immunologic characteristics.

**Results**: A total of 13 FRDEGs were obtained and eight were used to construct a diagnostic model and perform a prognostic analysis. Hub genes were also used to construct mRNA-miRNA and mRNA-TF interaction networks and potential drug or molecular compounds. Two MAFLD subtypes were identified: cluster2, which represents an “immunoactive” type, and cluster1, which represents an “immunosuppressive” type; a significant correlation was observed between the immune cell contents and the expression of three FRDEGs (*NR4A1*, *FADS2*, and *SCD*).

**Conclusion**: A ferroptosis-related gene signature was constructed to diagnose MAFLD-associated steatohepatitis, predict the prognosis of MAFLD patients, and analyze the immunologic characteristics of MAFLD. Our findings may provide insights into developing innovative MAFLD treatment techniques.

## 1 Introduction

Metabolic dysfunction-associated fatty liver disease (MAFLD), formerly known as non-alcoholic fatty liver disease (NAFLD), is a term that describes liver diseases associated with metabolic risk factors, such as obesity, Type 2 diabetes, and abnormal lipid metabolism (Eslam et al., 2020). The incidence and prevalence of MAFLD are rapidly increasing, with a prevalence of approximately 25% globally (Huang et al., 2021) and 42% in Asian countries (Li et al., 2019). MAFLD is a heterogeneous condition with complex and disparate causes that damages organs other than the liver. Pathophysiological changes associated with MAFLD range from simple steatosis to steatohepatitis, cirrhosis, and hepatocellular carcinoma. Most individuals with MAFLD are in the hepatic steatosis stage, whereas the minority show inflammation injury that can occur at any point (Eslam et al., 2020). Furthermore, hepatocyte ferroptosis may cause steatohepatitis and fatty liver deterioration (Chen et al., 2022). Currently, there are no pharmacologic treatments for MAFLD; therefore, non-pharmacologic interventions remain the mainstay of treatment for MAFLD. A novel approach to treating MAFLD may involve inhibiting hepatocyte ferroptosis.

Ferroptosis is a form of programmed cell death that differs from apoptosis and necrosis. It is iron dependent and associated with oxidative stress (Dixon et al., 2012), glutathione depletion, and glutathione peroxidase inactivation (Feng et al., 2022). The major molecular mechanism of ferroptosis has three key components: cystine absorption mediated by transport receptors of glutamate and cystine, named System x_c_-; glutathione depletion in response to iron and the inactivation of glutathione peroxidase 4 (GPx4) (Dixon et al., 2012); and high levels of lipid peroxidation, and intracellular reactive oxygen species build-up (Friedmann Angeli et al., 2014).

Ferroptosis has been extensively studied in neoplastic diseases; however, its research in MAFLD is limited. Some reviews have summarized the association between ferroptosis and MAFLD (Zhao et al., 2021; Feng et al., 2022; Ji et al., 2023). Furthermore, recent studies have shown that ferroptosis can cause an inflammatory response in simple steatosis, promoting the emergence and progression of steatohepatitis (Capelletti et al., 2020; Mao et al., 2020). In 2011, researchers found iron build-up in the livers of patients with MAFLD (Dongiovanni et al., 2011; Fujita and Takei, 2011; Salomao, 2021). Moreover, iron overload caused by metabolic dysfunctions, such as hepatosiderosis and hereditary hemochromatosis, could lead to iron accumulation in several tissues, especially the liver (Nelson et al., 2011; Partridge et al., 2014). In addition, iron removal could improve liver injury in patients with MAFLD (Valenti et al., 2011). Elevated levels of iron and lipid peroxidation markers, such as malondialdehyde and 4-hydroxynonenal, are present in patients with MAFLD (Loguercio et al., 2001). Vitamin E is a relatively effective suppressor of lipid peroxidation and could alleviate liver damage (Sanyal et al., 2010). However, the specific mechanism of ferroptosis in patients with MAFLD remains largely unclear.

In recent years, technological advancements in DNA/RNA sequencing and bioinformatics data analysis have increased our understanding of the mechanisms of health and disease. Some studies have investigated ferroptosis in MAFLD using bioinformatics. One study emphasized selenoprotein and selenium metabolism pathways (Day et al., 2021), and another identified two highly heterogeneous MAFLD subtypes with distinct clinical features, biological processes, and immune statuses based on ferroptosis-related gene expression (Dai et al., 2022).

In this study, our aim was to investigate ferroptosis in MAFLD using bioinformatics methods, including RNA-seq data analysis, consistent clustering, ssGSEA, *etc.* Our study highlights the important role of ferritin in the pathophysiology of MAFLD, particularly in its association with inflammation and fibrosis. This finding helps to deepen our understanding of the mechanisms underlying the development of MAFLD. Furthermore, we hypothesized that an in-depth analysis of the ferroptosis-related genes of MAFLD could be used to construct a diagnostic model and prognostic signature to explore immunologic characteristics and potential therapeutic drug targets, which might have a positive impact on improving the health of MAFLD patients.

## 2 Materials and methods

### 2.1 Data retrieval

The expression profile datasets, GSE48452 (Ahren et al., 2013), GSE63067 (Frades et al., 2015), and GSE89632 (Arendt et al., 2015), for patients with MAFLD, were downloaded from the Gene Expression Omnibus (GEO) database (Barrett et al., 2007) using the R package, GEOquery (Davis and Meltzer, 2007). GSE48452, GSE63067, and GSE89632 were all obtained from *Homo sapiens*. The GSE48452 dataset was derived from human liver samples. The inclusion criteria were: patients aged 18 years and having undergone a liver biopsy-based diagnosis of MAFLD, including simple hepatic steatosis and steatohepatitis.

The data platform for the GSE48452 dataset was GPL11532 [HuGene-1_1-st] Affymetrix Human Gene 1.1 ST Array [transcript (gene) version], whereas that for the GSE63067 dataset was GPL570 [HG-U133_Plus_2] Affymetrix Human Genome U133 Plus 2.0 Array, and the data platform for the GSE89632 dataset is GPL14951 Illumina HumanHT-12 WG-DASL V4.0 R2 expression beadchip. The dataset probe names were all annotated using the ChIP GPL platform files.

We collected ferroptosis-related genes from multiple database sources using the term “Ferroptosis” as the search keyword. The GeneCards database (Stelzer et al., 2016) (https://www.genecards.org/) provided comprehensive information on human genes. Furthermore, we obtained more genes from the FerrDb database (Zhou and Bao, 2020). The final sample was obtained after combined deduplication, as described in Supplementary Table S1.

### 2.2 Differentially expressed genes (DEGs) associated with MAFLD

We merged the MAFLD datasets GSE48452 and GSE63067 to identify the DEGs associated with MAFLD, their underlying mechanisms of action, related biological features, and disease pathways. Subsequently, we removed the batch effects using the R surrogate variable analysis package and standardized the combined dataset using the limma package (Ritchie et al., 2015). Thereafter, we obtained the MAFLD dataset and the expression matrix of the principal component analysis (PCA) (Ben Salem and Ben Abdelaziz, 2021). PCA is the preferred method for reducing data dimension and analyzing the effect of batch effect removal. The feature vectors of the data were extracted from high-latitude data, converted to low-dimensional data, and displayed with two-dimensional or three-dimensional graphs.

We used the limma package on the MAFLD dataset to obtain the DEGs between different groups within the MAFLD dataset. The genes selected by | logFC | > 0.5 and *p* < 0.05 were used as our DEGs. genes with logFC >0.5 and *p* < 0.05 were DEGs with upregulated expression (upregulated genes), and those with logFC < −0.5 and *p* < 0.05 were DEGs with downregulated expression (downregulated genes).

To obtain FRDEGs associated with MAFLD, we crossed the MAFLD dataset DEGs with | logFC |> 0.5 and *p* < 0.05 and the ferroptosis-related genes and then drew a Venn diagram. The results of the differential analysis were illustrated with the volcano map using the R package ggplot2 and the heatmap map using the R package pheatmap.

### 2.3 Receiver operating characteristic (ROC) curve

The ROC curve (Mandrekar, 2010) is an analysis tool for a coordinate scheme that can be used to select the best model, discard the next best model, or set the best threshold in the same model. The ROC curve is a comprehensive index of continuous variables that reflect sensitivity and specificity. Reflects the interrelationship between sensitivity and specificity through the composition method. The area value under the ROC curve (AUC) is between 0.5 and 1. We used the R survival ROC package to plot the ROC curve of FRDEGs in the MAFLD dataset. The AUC was calculated to evaluate the diagnostic effect of FRDEG expression on the survival of patients with MAFLD. The diagnostic effect is better if the AUC value is closer to 1. AUC values of 0.5–0.7, 0.7–0.9, and >0.9 indicate low, moderate, and high diagnostic accuracy, respectively.

### 2.4 Differential functional gene enrichment analysis and pathway enrichment analysis

Gene ontology (GO) analysis (Yu, 2020) is a common method for conducting large-scale functional enrichment studies, including those to identify the biological processes (BPs), molecular functions (MFs), and cellular components (CCs) for which genes are enriched. The Kyoto Encyclopedia of Genes and Genomes (KEGG) (Kanehisa and Goto, 2000) is a database widely used to store information about genomes, biological pathways, diseases, and drugs. The clusterProfiler (Yu et al., 2012) of the R package was used to perform the GO annotation analysis of FRDEGs, and the entry screening criteria were *p* < 0.05 and a false discovery rate (FDR) value (q-value) < 0.05, which was considered statistically significant. The *p*-value was corrected using the Benjamini–Hochberg (BH) method.

### 2.5 Gene set enrichment analysis (GSEA)

GSEA is used to assess the distribution trend of genes in a predefined gene set in a list of genes ranked by phenotype correlation to determine their contribution to the phenotype (Subramanian et al., 2005). In this study, genes in the MAFLD dataset were divided into two groups according to their phenotype correlations. Subsequently, we used the clusterProfiler package to enrich all DEGs in phenotype correlation. The parameters used in the GSEA were as follows: seed 2021, 1,000 calculations, 10 genes per gene set, and a maximum of 500 genes. Correction of the *p-value* was performed using the BH method. We obtained the c2. cp.v7.2. symbols gene set from the Molecular Signatures Database (MSigDB); significantly enriched screening criteria were *p* < 0.05 and FDR value (q-value) < 0.05.

### 2.6 Constructing a FRDEG-related diagnostic model

To obtain a diagnostic model for FRDEGs in the MAFLD dataset, we used the glmnet package (Engebretsen and Bohlin, 2019) based on the FRDEGs with the set of parameters: seed (2), family = “binomial” to perform the least absolute shrinkage and selection operator (LASSO) regression and run 1,000 cycles to prevent overfitting. LASSO regression is often used to build prognostic models. Model overfitting was reduced, and the model’s generalization ability was improved by increasing the penalty term (the absolute value of lambda slope) based on linear regression. We visualized the LASSO regression results and presented the molecular expression of each gene in the FRDEG diagnostic model using forest plots. The FRDEGs screened by LASSO regression were subjected to multivariate Cox regression analysis and were constructed into multivariate Cox regression models.

Subsequently, we constructed a nomogram based on the results of the multivariate Cox regression analysis (Park, 2018). A nomogram is a graph based on multivariate regression analysis, and by setting a certain scale to score the variables in the multivariate regression model, it can be used to predict the probability of events by calculating the total score. Decision curve analysis (DCA) (Van Calster et al., 2018) is a simple method for evaluating clinical predictive models, diagnostic tests, and molecular markers. Finally, we evaluated the accuracy and resolution of the Cox regression model by drawing DCA plots using the ggDCA R package (Tataranni and Piccoli, 2019).

### 2.7 Protein–protein interaction (PPI) network

PPI networks comprise individual proteins involved in biological signaling, gene expression regulation, energy and substance metabolism, and cell cycle regulation. The STRING database (Szklarczyk et al., 2019) can be used to search for known proteins and predict interactions between them. In this study, the STRING database was used to construct a PPI network (minimum interaction score required: medium confidence [0.150]) using LASSO regression. Cytoscape (Shannon et al., 2003; Cytoscape consortium version 3.9.1 was downloaded from https://cytoscape.org/) was used to visualize the PPI network model and select the FRDEGs associated with other nodes as the key genes (hub genes) of MAFLD.

### 2.8 Construction of mRNA-miRNA, mRNA-transcription factor, and mRNA-drug interaction networks

The Encyclopedia of RNA Interactomes (ENCORI) (Li et al., 2014) database (https://starbase.sysu.edu.cn) is version 3 h.0 of the starBase database. The ENCORI database for the interactions of miRNA-ncRNA, miRNA-mRNA, ncRNA-RNA, RNA-RNA, RBP-ncRNA, and RBP-mRNA is based on CLIP-seq, degradome sequencing (for plants) and data mining, providing various visual interfaces for exploring microRNA targets. The miRDB (Liberzon et al., 2015; Chen and Wang, 2020) is a database for miRNA target gene prediction and functional annotation. The ENCORI and miRDB were used to predict the miRNAs that would interact with hub genes, and the mRNA-miRNA interaction network was constructed after crossing the data section of target score >80 in the miRDB database with the mRNA-miRNA data in the ENCORI database.

The CHIPBase database (Zhou et al., 2017) (version 2.0, https://rna.sysu.edu.cn/chipbase/) has identified thousands of binding motif matrices and their binding sites from the ChIP-seq data for DNA-binding proteins and predicted millions of regulatory transcriptional relationships between transcription factors (TF) and genes. The hTFtarget database (Zhang et al., 2020) (http://bioinfo.life.hust.edu.cn/hTFtarget) contains data on human TF and their corresponding regulatory targets. TFs bound to hub genes were searched through the CHIPBase and hTFtarget databases and visualized using Cytoscape software.

The Public Comparison Toxics Genomics Database (Comparative Toxicogenomics Database, CTD (Davis et al., 2021), http://ctdbase.org/) is a database based on innovative digital ecosystems that link chemicals, genes, phenotypes, diseases, and known toxicology information to facilitate access to human health-related data. The CTD was also used to predict potential drugs or small molecule compounds that would interact with hub genes, and mRNA-miRNA, mRNA-TF, and mRNA-drug interaction networks were visualized using Cytoscape software.

### 2.9 Construction of disease subtypes based on FRDEGs

Consensus clustering (Brière et al., 2021) is a resampling-based algorithm used to identify each member and its subgroup number and to verify clustering. Consensus clustering involves multiple iterations on subsamples of datasets that provide indicators of clustering stability and parameter decisions by exploiting subsampling and inducing sampling variability. The consensus clustering method of the ConsensusClusterPlus package in R (Wilkerson and Hayes, 2010) was used to identify different MAFLD disease subtypes based on FRDEGs.

### 2.10 Identification and correlation analysis of immune-infiltrating cells among different MAFLD disease subtypes

The single-sample GSEA (ssGSEA) algorithm was used to quantify the relative abundance of each immune cell infiltrate. Various infiltrating immune cell subtypes, such as activated CD8^+^ T cells, activated dendritic cells, macrophages, natural killer T cells, and regulatory T cells, were labeled. Enrichment scores calculated by ssGSEA were used to represent the relative abundance of each immune cell infiltrate in each sample (Barbie et al., 2009; Charoentong et al., 2017). The ggplot2 package was used to visualize the differential expression relationship of immune cells in the different disease subtypes in the MAFLD dataset.

The degree of the infiltration of tumor microenvironment, immune cells, and stromal cells in tumors significantly affects the prognosis. The ESTIMATE package (Yoshihara et al., 2013) uses the unique transcriptional profiles of cancer samples to infer the content of tumor cells and different infiltrating normal cells. To better understand the prognostic impact of genes involved in immune and stromal cells, the ESTIMATE package was used to calculate the immune and mechanism scores of the different disease subtype samples from the MAFLD dataset and to assess the purity of the tumor. The principle is based on the ESTIMATE algorithm to quantify the immune and stromal components in the tumors by calculating the ESTIMATE, Immune, and Stromal scores, and purity of the tumor expression matrix characteristics.

### 2.11 Differential analysis of FRDEG expression in clinical subgroups

To explore the expression differences of 13 FRDEGs in the GSE48452 of the MAFLD dataset neutron dataset, Mann‒Whitney U test (Wilcoxon rank sum test) was used to combine the specific clinical characteristics grouping information (sex, age, BMI, leptin, and adiponectin) and to analyze the expression level of 13 FRDEGs in GSE48452 dataset samples in the MAFLD dataset and the expression difference between different groups (numerical grouping was not strictly performed using median values, but by the range of values, with each sample equal). We showed the results of the expression difference analysis through a group comparison diagram.

### 2.12 Cell culture and treatment

For *in vitro* research, Procell Life Science&Technology (Wuhan, Hubei, China) supplied mouse hepatocytes AML12. AML12 cells were grown in Dulbecco’s modified Eagle medium (DMEM) at 37 °C in a humid environment under 5% CO_2_ conditions; the medium was supplemented with 10% (v/v) fetal bovine serum 1% penicillin/streptomycin. The steatohepatitis cell model (SHC) was established by administering 0.25 mmol/L palmitic acid (PA) and 0.5 mmol/L oleic acid (OA) to the cells for 24 h, followed by culturing in methionine-choline and glutamine-deficient (MCD) DMEM.

### 2.13 Reverse transcription-quantitative polymerase chain reaction (RT-qPCR)

Total RNA was extracted using the Trizol reagent and the RT kits for reverse transcription were provided by TOYOBO (Japan). The SYBR Green qRT-PCR Mix (Solarbio, Beijing, China) was used to perform RT-qPCR. Using 2^−ΔΔCT^, the relative expression was estimated with *GAPDH* as the internal reference. The following are the forward and reverse primers: *NR4A1* (F: 5′-CCG​TGG​CTT​TGG​TGA​TTG​GAT​TG′-3′, R: 5′-TGA​GGA​CCA​GAG​CGG​ACA​GG-3); *FADS2* (F: 5′-AGA​AGA​CTG​CTG​AGG​ACA​TGA​ACC′-3′, R: 5′-CGA​GAG​GAT​GAA​CCA​GGC​AAG​G-3′); stearoyl-CoA desaturase (*SCD*) (F: 5′-TGA​GGC​GAG​CAA​CTG​ACT​ATC​ATC′-3′, R: 5′-TGG​TGG​TGG​TCG​TGT​AAG​AAC​TG-3′); *GAPDH* (F: 5′-GCA​TCC​ACT​GGT​GCT​GCC-3′, R: 5′-TCA​TCA​TAC​TTG​GCA​GGT​TTC-3′).

### 2.14 Statistical analysis

All the data processing used for the bioinformatics analysis in this study were performed using the R software (Version 4.1.2). For continuous variables, data are presented as the mean ± standard deviation; distributed data are presented as median. Comparisons between two groups were made using the Wilcoxon rank sum test, whereas comparisons between three or more groups were made using the Kruskal–Wallis test. The chi-square or Fisher’s exact test was used to compare and analyze statistical significance between two groups of categorical variables. If not indicated explicitly, all results were all calculated as correlation coefficients between different molecules using Spearman correlation analysis, and all results used *p* < 0.05 as the criterion for significant difference results.

GraphPad Prism (GraphPad Prism Software, CA, United States) was used to analyze the PCR data. Student’s unpaired *t*-test (two-group comparisons) was performed and *p* < 0.05 was considered statistically significant.

## 3 Results

### 3.1 Technical roadmap

#### 3.1.1 Extracted data

A total of 73 cases in the GSE48452 dataset were obtained from the GEO database, including 14 normal control samples and 59 human liver biopsy samples of different phases from patients with MAFLD. The MAFLD group included liver biopsy samples from 27 cases of healthy patients with obesity, 14 cases with simple hepatic steatosis, and 18 cases with steatohepatitis, whereas the microarray GSE63067 dataset included gene expression profiles of 11 human liver samples from patients with MAFLD (including two cases with simple hepatic steatosis and nine cases with steatohepatitis) and seven healthy individuals. The samples for the GSE89632 dataset were human liver samples, totaling 63 cases, including 24 normal samples, 39 human liver samples from patients with MAFLD (20 patients with steatosis, and 19 patients with steatohepatitis).

Subsequently, we analyzed the data profile of 32 cases from the GSE48452 dataset, including 18 liver biopsy samples from patients with steatohepatitis (group: MAFLD) and 14 normal samples (group: Control). A total of 16 expression profiles were analyzed in the GSE63067 dataset, including those of nine liver biopsy samples from patients with steatohepatitis (group: MAFLD) and seven healthy human liver samples (group: Control). A total of 43 expression profile data were analyzed in the GSE89632 dataset, including those of 19 liver biopsy samples from patients with steatohepatitis (group: MAFLD) and 24 healthy human liver samples (group: Control). The GSE89632 dataset was used as a validation set to verify the expression of genes in different groups (Control/MAFLD). The specific dataset information is presented in Table 1.

**TABLE 1:** GEO microarray Chip information.

	GSE48452	GSE63067	GSE89632
Platform	GPL11532	GPL570	GPL14951
Species	*Homo sapiens*	*Homo sapiens*	*Homo sapiens*
Tissue	Human liver biopsy samples	Human liver samples	Human liver samples
Samples in the MAFLD group	18	9	19
Samples in the control group	14	7	24
References	PMID: 23931760	PMID: 25993042	PMID: 25581263

GEO: gene expression omnibus.

We identified 619 and 567 ferroptosis-related genes from the GeneCards and FerrDb databases, respectively. After combined deduplication (Supplementary Table S1), 958 ferroptosis-related genes were obtained for analysis.

### 3.2 MAFLD metabolism-related differential gene analysis

**FIGURE 1:**
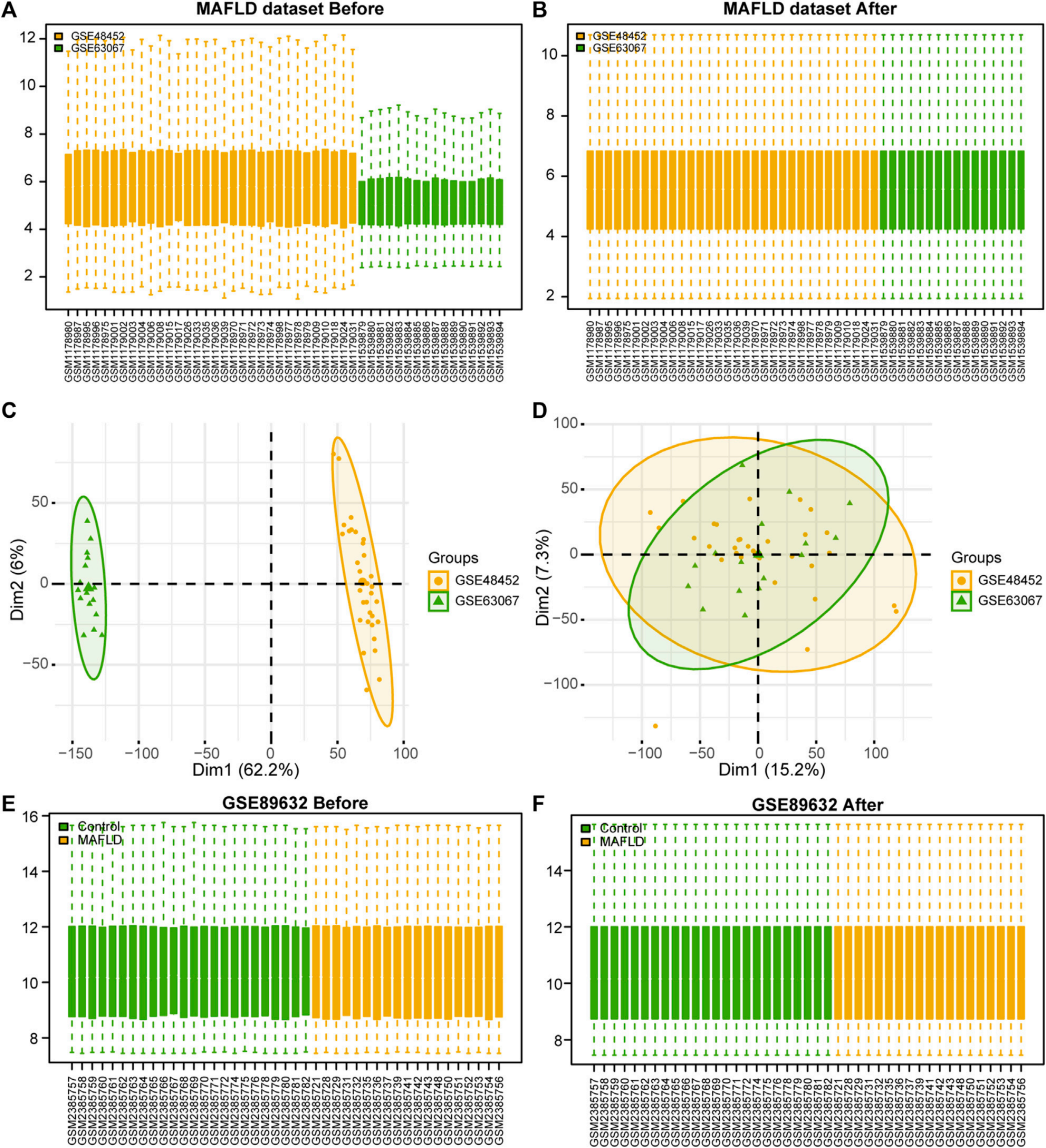
MAFLD dataset de-batch processing. **(A, B)** MAFLD dataset removes the batch effect. **(A)** The box plot before processing. **(B)** The boxplot plot after processing. **(C, D)** NAFLD dataset removes the batch effect. **(C)** The PCA plot before processing. **(D)** The PCA plot after processing. **(E, F)** Box plot of normalized processing before **(E)** and after **(F)** of the GSE89632 dataset. MAFLD: metabolic dysfunction-associated fatty liver disease; PCA: principal component analysis.

After removing batch effects on the two MAFLD datasets and standardizing the combined dataset, we obtained the overall MAFLD dataset (Figures 1A, B). The MAFLD dataset included 27 steatohepatitis samples (group: MAFLD) and 21 healthy samples (group: Control). The results of the PCA performed on the dataset expression matrix before and after batch effect removal confirmed its elimination in the MAFLD dataset (Figures 1C, D).

We standardized the GSE89632 dataset using the limma package in R to obtain the standardized GSE89632 dataset (Figures 1E, F). The results showed that the batch effect of samples in the MAFLD dataset was also eliminated after the standardization treatment.

**FIGURE 2:**
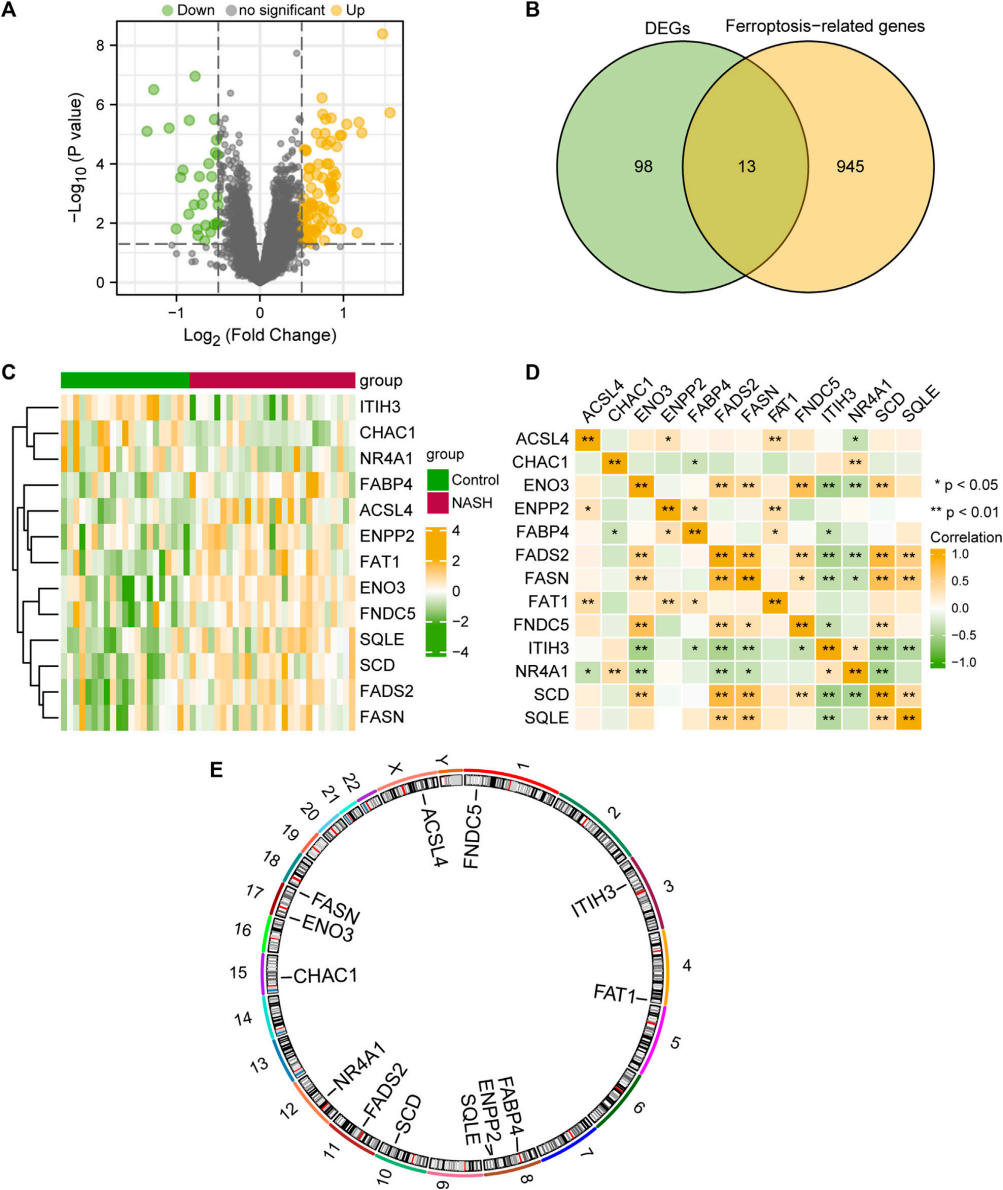
Differential gene expression analysis of the MAFLD dataset. **(A)** MAFLD dataset volcano plot of differential expression gene analysis in the steatohepatitis (group: MAFLD) and Control (group: Control) groups. Green represents downregulated genes and yellow represents upregulated up-regulated genes **(B)** Venn diagram of DEGs and ferroptosis-related genes in the MAFLD dataset. **(C)** Complex numerical heatmap of FRDEGs in the MAFLD dataset. Yellow represents positive correlation and green represents positive correlation **(D)** Correlation analysis of the FRDEGs in the MAFLD dataset. Yellow represents positive correlation and green represents positive correlation **(E)** Chromosome localization map of the FRDEGs. MAFLD: metabolic dysfunction-associated fatty liver disease; FRDEGs: ferroptosis-related differentially expressed genes; DEGs: differentially expressed genes. ns, *p* ≥ 0.05, not significant; *p < 0.05, significant; **p < 0.01, highly significant; and ***p < 0.001, very highly significant.

To analyze the differences in gene expression within the MAFLD group compared to those of the Control group, we used the limma package to obtain the DEGs among different groups in the MAFLD dataset. The results were as follows: among the 15,370 DEGs in the MAFLD dataset, 111 genes met the | logFC |> 0.5 and the *p-*value <0.05 thresholds. At this threshold value, the number of upregulated genes in the MAFLD group (identified by low expression in the Control group, positive logFC) was 82, whereas the number of downregulated genes in the MAFLD group (high expression in the Control group, negative logFC) was 29. We created a volcano map to illustrate the results of the MAFLD dataset (Figure 2A).

To obtain FRDEGs, we recorded the intersection of all DEGs with | logFC |> 0.5 and *p* < 0.05 obtained in the MAFLD dataset. A total of 13 FRDEGs for MAFLD were obtained and illustrated with a Venn diagram (Figure 2B). The 13 FRDEGs were acyl-CoA synthetase long-chain family member 4 (*ACSL4*), ChaC glutathione specific gamma-glutamylcyclotransferase 1 (*CHAC1*), enolase 3 (*ENO3*), ectonucleotide pyrophosphatase/phosphodiesterase 2 (*ENPP2*), fatty acid binding protein 4 (*FABP4*), fatty acid desaturase 2 (*FADS2*), fatty acid synthase (*FASN*), FAT atypical cadherin 1 (*FAT1*), fibronectin Type III domain containing 5 (*FNDC5*), inter-alpha-trypsin inhibitor heavy chain 3 (*ITIH3*), nuclear receptor subfamily 4 group A member 1 (*NR4A1*), *SCD*, and squalene epoxidase (*SQLE*). Specific gene information is presented in Table 2.

**TABLE 2:** Ferroptosis-related differentially expressed gene information list.

	Gene symbol	Description	Category	logFC	*p*-value	AveExpr
1	*ACSL4*	Acyl-CoA Synthetase Long-Chain Family Member 4	Protein Coding	0.823464	0.003938	5.828833
2	*CHAC1*	ChaC Glutathione Specific Gamma-Glutamylcyclotransferase 1	Protein Coding	−0.788984	0.002374	4.913718
3	*ENO3*	Enolase 3	Protein Coding	1.221403	0.000009	7.313875
4	*ENPP2*	Ectonucleotide Pyrophosphatase/Phosphodiesterase 2	Protein Coding	0.531732	0.020691	7.975373
5	*FABP4*	Fatty Acid Binding Protein 4	Protein Coding	0.887550	0.001471	5.331615
6	*FADS2*	Fatty Acid Desaturase 2	Protein Coding	0.883478	0.000177	8.214937
7	*FASN*	Fatty Acid Synthase	Protein Coding	0.594994	0.019310	8.275047
8	*FAT1*	FAT Atypical Cadherin 1	Protein Coding	0.753564	0.000293	7.632159
9	*FNDC5*	Fibronectin Type III Domain Containing 5	Protein Coding	0.758143	0.004707	7.124574
10	*ITIH3*	Inter-Alpha-Trypsin Inhibitor Heavy Chain 3	Protein Coding	−0.521859	0.000016	11.171580
11	*NR4A1*	Nuclear Receptor Subfamily 4 Group A Member 1	Protein Coding	−0.650639	0.011771	6.054939
12	*SCD*	Stearoyl-CoA Desaturase	Protein Coding	0.850099	0.000569	8.355172
13	*SQLE*	Squalene Epoxidase	Protein Coding	0.864208	0.000283	7.042331

We analyzed the differential expression of 13 FRDEGs among different groups within the MAFLD dataset according to the results obtained by the Venn diagram and used the heatmap constructed with the pheatmap package in R to visualize the specific differential analysis results (Figure 2C). Subsequently, we showed the correlations between the expression of 13 FRDEGs in the MAFLD dataset and that in the heatmap (Figure 2D). In addition, we annotated the 13 FRDEGs using the RCircos package (Figure 2E) to analyze the chromosomal locations of the 13 FRDEGs. The FRDEGs were mainly distributed on chromosomes 3, 4, 8, 10, 11, and 12, with the largest distribution on chromosome 8 (three FRDEGs).

### 3.3 Differential expression analysis and validation of the FRDEGs

**FIGURE 3:**
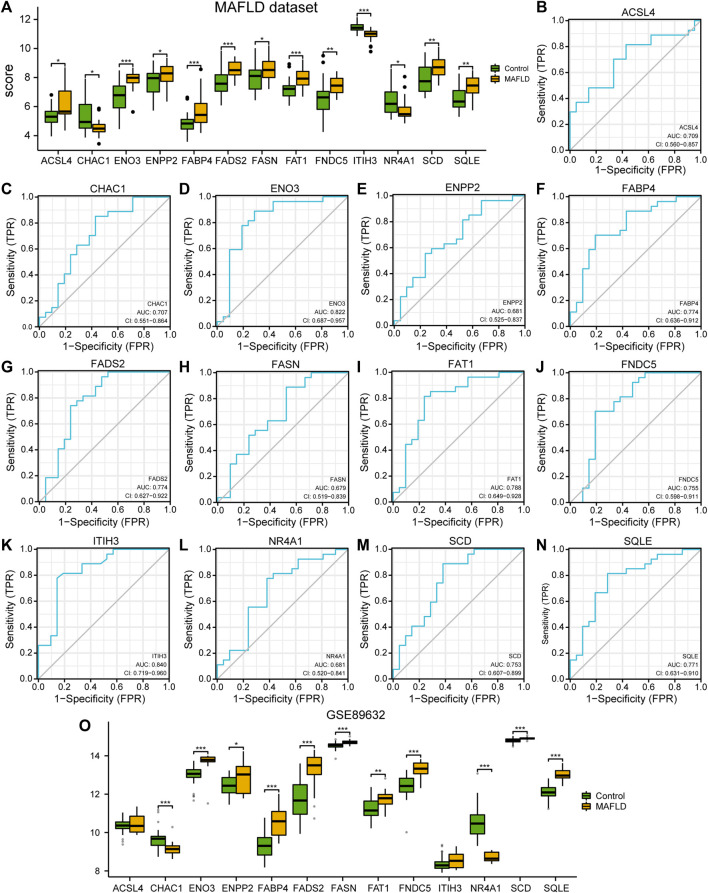
Differential analysis of FRDEG expression and verification in the MAFLD dataset. **(A)** Group comparison plot of the FRDEG expression difference analysis in the MAFLD dataset. (B–N) The ROC curves for the FRDEGs *ACSL4* **(B)**, *CHAC1* **(C)**, *ENO3* **(D)**, *ENPP2* **(E)**, *FABP4* **(F)**, *FADS2* **(G)**, *FASN* **(H)**, *FAT1* **(I)**, *FNDC5* **(J)**, *ITIH3* **(K)**, *NR4A1* **(L)**, *SCD* **(M)**, and *SQLE* **(N)** in the MAFLD dataset. **(O)** Group comparison diagram of the differences the expression levels of FRDEGs in the GSE89632 dataset. ns, *p* ≥ 0.05, not significant; *p < 0.05, significant; **p < 0.01, highly significant; and ***p < 0.001, very highly significant. MAFLD: metabolic dysfunction-associated fatty liver disease; FRDEGs: ferroptosis-related differentially expressed genes; ROC: receiver operating characteristic curve.

To explore the differential expression of the 13 FRDEGs in the MAFLD dataset, we used the Mann–Whitney U test (Wilcoxon rank sum test) to analyze the relationship between the expression levels of the 13 FRDEGs and the different groups (MAFLD groups/Control groups). We showed the correlation analysis results using group comparison plots (Figure 3A). As shown in Figure 3A, the expression of all 13 FRDEGs in the MAFLD dataset differed significantly among the groups (*p <* 0.05). The expression levels of *ENO3*, *FABP4*, *FADS2*, *FAT1*, and *ITIH3* in the MAFLD dataset showed very highly significant differences among the different groups (*p <* 0.001). Furthermore, the expression levels of *FNDC5*, *SCD*, and *SQLE* in the MAFLD dataset showed highly significant differences among the different groups (*p <* 0.01).

Subsequently, we drew the ROC curves for the 13 FRDEGs in the MAFLD dataset (Figure 3B–N). The ROC curve results were as follows: the expression levels of FRDEGs *ENPP2* (AUC = 0.681, Figure 3E), *FASN* (AUC = 0.679, Figure 3H), and *NR4A1* (AUC = 0.681, Figure 3L) in the MAFLD dataset showed a low correlation between the different groups. The expression levels of the ACSL4 genes (AUC = 0.709, Figure 3B), *CHAC1* (AUC = 0.707, Figure 3C), *ENO3* (AUC = 0.822, Figure 3D), *FABP4* (AUC = 0.774, Figure 3F), *FADS2* (AUC = 0.774, Figure 3G), *FAT1* (AUC = 0.788, Figure 3I), *FNDC5* (AUC = 0.755, Figure 3J), *ITIH3* (AUC = 0.840, Figure 3K), *SCD* (AUC = 0.753, Figure 3M) and *SQLE* (AUC = 0.771, Figure 3N) showed a certain correlation between the MAFLD and Control groups in the MAFLD dataset.

Finally, we analyzed the correlation between expression level of 13 FRDEGs in the GSE89632 dataset and the different groups (MAFLD group/Control group); we showed the correlation analysis results using a group comparison diagram (Figure 3O). The results showed that the expression of 13 FRDEGs in the GSE89632 dataset, except for *ACSL 4* and *ITIH 3*, differed significantly between the groups (*p <* 0.05). Among these genes, the expression levels of *CHAC 1, ENO3, FABP4, FADS2, FASN, FNDC5, NR4A1, SCD,* and *SQLE* in the GSE89632 dataset showed very highly significant differences among the different groups (*p <* 0.001); moreover, the expression levels of the genes *ENPP2* and *FAT1* in the GSE89632 dataset showed very significant differences among the different groups (*p <* 0.01).

### 3.4 GO and KEGG enrichment analyses of FRDEGs

**FIGURE 4:**
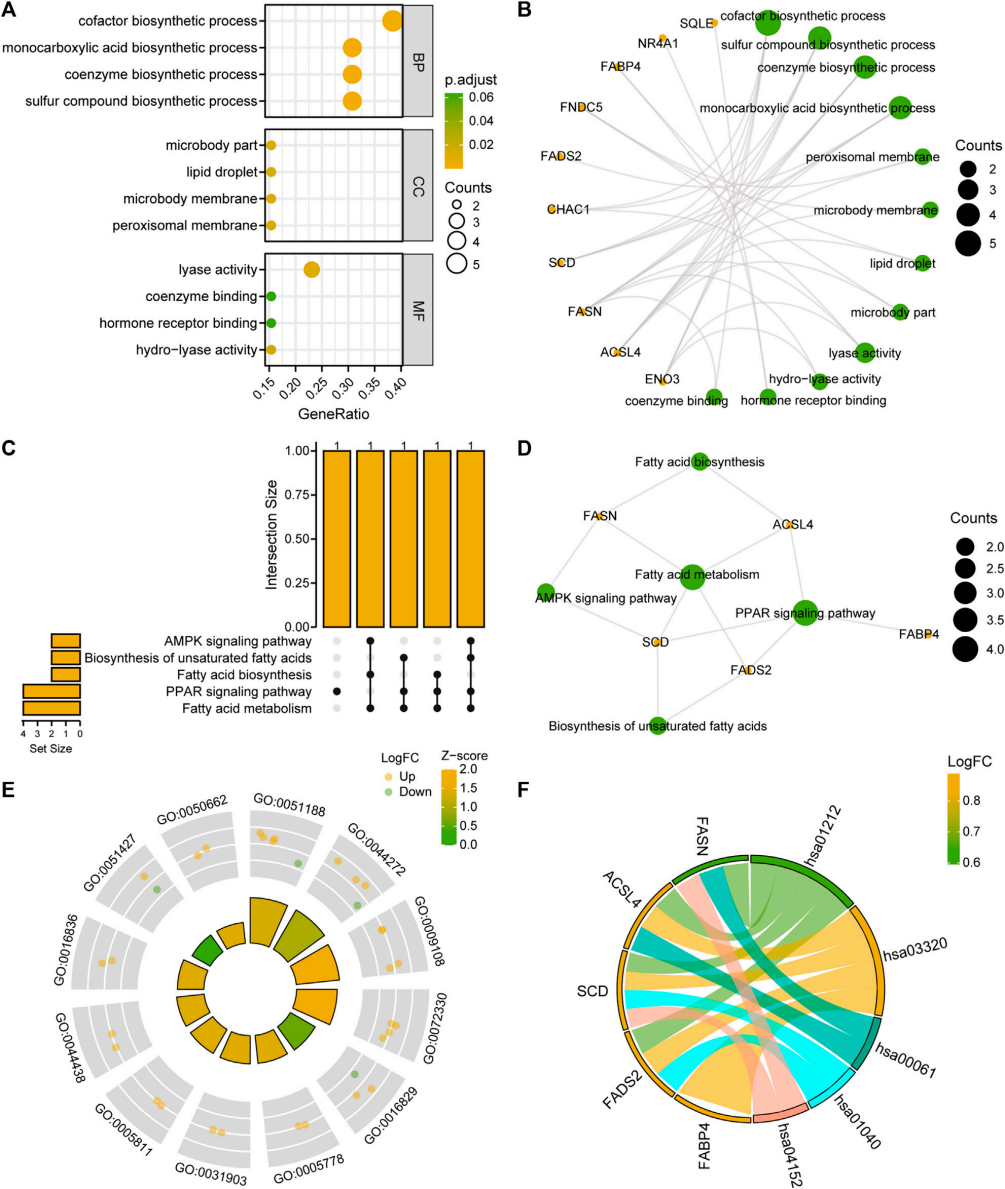
FRDEG functional enrichment analysis (GO) and pathway enrichment (KEGG) analysis. **(A, B)** Results of the GO function enrichment analysis of the FRDEGs are displayed as **(A)** a bubble diagram and **(B)** a ring network diagram. **(C, D)** Results of the KEGG pathway enrichment analysis results of the FRDEGs are displayed as **(C)** an upset diagram and **(D)** a network diagram. **(E)** Circle plot display of the GO function enrichment with combined logFC analysis results for the FRDEGs. **(F)** Chord plot presentation of the KEGG pathway enrichment analysis combined with the logFC analysis results for the FRDEGs. The coordinates in the bubble chart **(A)** represent the GO terms; the bubble color indicates the GO terms showing activation or inhibition; the orange yellow color indicates activation and the light-green color indicates inhibition. The orange dots in the network diagrams **(B, D)** represent specific genes and the light-green circles represent specific pathways. The circle **(E)** represents the upregulated genes (logFC >0), and the light green dots represent the downregulated genes (logFC <0). FRDEGs: ferroptosis-related differentially expressed genes; GO: Gene Ontology; BP: biological process; CC: cellular component; MF: molecular function; KEGG: Kyoto Encyclopedia of Genes and Genomes. The screening criteria for the GO and KEGG enrichment entries were the *p* < 0.05 and FDR value (q-value) < 0.05.

To analyze the BPs, MFs, and CCs that were enriched for the 13 FRDEGs and the relationship between biological pathways and NAFLD, we performed a GO functional enrichment analysis (Supplementary Table S2) of the FRDEGs. The screening criteria for the enrichment entries were *p* < 0.05; FDR values (q-values) < 0.05 were considered significant. The results showed that the 13 FRDEGs in MAFLD were mainly enriched for the following BPs: biosynthesis of cofactors, sulfur compounds, coenzymes, and monocarboxylic acid; the following CCs: the peroxisomal and microbody membranes, lipid droplets, and microbody parts; and the following MFs: lyase activity, hydro-lyase activity, hormone receptor binding, and coenzyme binding. We have presented the results of the GO enrichment analysis in a bubble diagram (Figure 4A) and a circular network map (Figure 4B).

We performed a KEGG enrichment analysis (Supplementary Table S3) of the 13 FRDEGs. The results indicated that the 13 FRDEGs were significantly enriched in five KEGG pathways, such as fatty acid metabolism, the peroxisome proliferator-activated receptor (PPAR) signaling pathway, fatty acid, and unsaturated fatty acid biosynthesis, and the AMPK signaling pathway. We used upset maps (Figure 4C) and network maps (Figure 4D) to show the gene expression in the five KEGG pathways. Subsequently, we performed GO and KEGG enrichment analyses of the joint logFC for these 13 FRDEGs based on the enrichment analysis by providing the logFC value of the genes of different analysis results in the MAFLD dataset to calculate a z-score for each gene. We have presented the GO enrichment analysis results of the joint logFC as a circle diagram (Figure 4E) and the KEGG enrichment analysis results of the joint logFC as a chord diagram (Figure 4F).

### 3.5 GSEA of the MAFLD dataset

**FIGURE 5:**
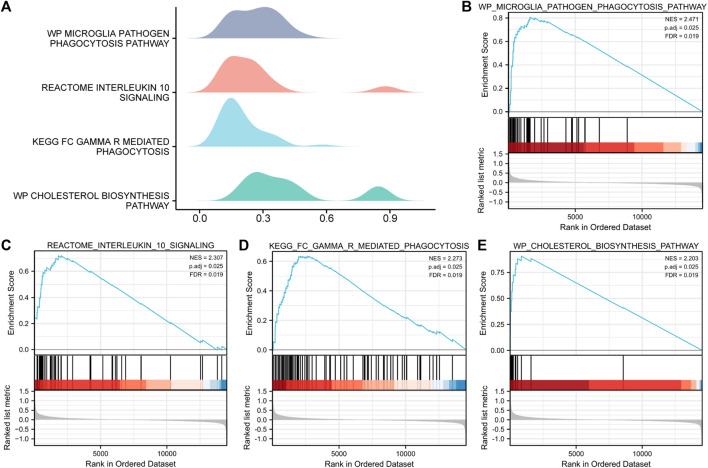
GSEA of the MAFLD dataset. **(A)** GSEA of the MAFLD dataset. **(B–E)** The DEGs in the MAFLD dataset were significantly enriched in **(B)** the microglia pathogen phagocytosis pathway, **(C)** interleukin-10 signaling, **(D)** FcγR-mediated phagocytosis, and **(E)** cholesterol biosynthesis pathway. MAFLD: metabolic dysfunction-associated fatty liver disease; DEGs: differentially expressed genes; GSEA: gene set enrichment analysis. The screening criteria for significant enrichment for GSEA were *p* < 0.05 with an FDR value (q-value) < 0.05.

To determine the effects of the gene expression levels on the occurrence of MAFLD, we analyzed the expression of the genes in the MAFLD datasets and the BPs, CCs, and MFs enriched for these genes by GSEA (Figure 5A). *p* < 0.05 and FDR values (q-values) < 0.05 were the screening criteria for significant enrichment. The results showed that the expression levels of genes in the MAFLD dataset were significantly enriched in the microglia pathogen (Figure 5B), interleukin-10 signaling (Figure 5C), FcγR-mediated phagocytosis (Figure 5D), and cholesterol biosynthesis pathways (Figure 5E) (Figures 5A–E; Supplementary Table S4).

### 3.6 Diagnostic and prognostic performance of the FRDEG model

**FIGURE 6:**
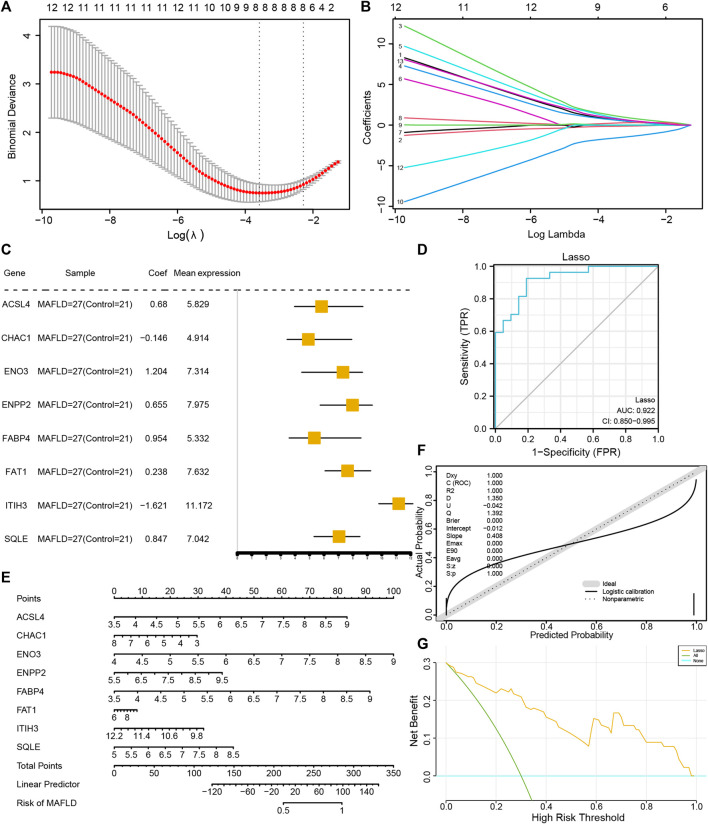
Construction of the FRDEG diagnostic model and its prognostic performance. **(A)** The least absolute shrinkage and selection operator (LASSO) binary logistic regression model used for constructing the FRDEG diagnostic model of MAFLD. The parameter set: seed (2), family = “binomial” performs the LASSO regression and runs for 1,000 cycles. **(B)** Ten-time cross-validation for tuning parameter selection in the LASSO model. **(C)** Forest plot of FRDEGs in the FRDEG diagnostic model. **(D)** The ROC curve of the FRDEG diagnostic model in the MAFLD dataset. **(E)** Nomogram plot, **(F)** calibration curve plot, and **(G)** DCA plot of the FRDEG univariate and multivariate Cox regression models in the FRDEG diagnostic model. In the DCA figure, the *X*-axis represents the probability threshold or the threshold probability and the *Y*-axis represents net income. The results could be judged by observing the x value range of the line of All positive and the line of All negative. The larger the x value range, the better the model effect. FRDEGs: ferroptosis-related differentially expressed genes; MAFLD: metabolic dysfunction-associated fatty liver disease; ROC: receiver operating characteristic curve; DCA: decision curve analysis.

To determine the diagnostic value of the 13 FRDEGs in the MAFLD dataset, we used LASSO regression analysis to construct a FRDEG diagnostic model (Figure 6A). LASSO regression was based on linear regression by increasing the penalty term (the absolute value of the lambda slope) and improving the model’s generalization ability. We also visualized the LASSO regression results and obtained the LASSO variable trajectory map (Figure 6B). The figure shows that we used eight FRDEGs, namely, *ACSL4*, *CHAC1*, *ENO3*, *ENPP2*, *FABP4*, *FAT1*, *ITIH3*, and *SQLE*. Next, we visualized the expression of the eight FRDEGs in the FRDEG diagnostic model in different groups using a forest map (Figure 6C). The coefficients of the eight genes based on the LASSO analysis were multiplied by the expression of the corresponding genes and summed to establish a MAFLD prediction score. We calculated the final prediction score for each sample and then drew its ROC curve using the prediction score. The results showed that the FRDEG diagnostic model could accurately predict MAFLD (AUC = 0.922, Figure 6D).

We performed uni- and multivariate Cox regression analyses of the eight FRDEGs (*ACSL4*, *CHAC1*, *ENO3*, *ENPP2*, *FABP4*, *FAT1*, *ITIH3*, and *SQLE*) and constructed a Cox regression model. Next, we performed a nomogram analysis to determine the prognostic ability of the model and drew a nomogram (Figure 6E); thereafter, we performed a prognostic calibration analysis of the nomograms of the univariate and multivariate Cox regression models and calibration curves (Figure 6F). Finally, we used DCA to evaluate the role of the constructed Cox regression model in clinical practice. The results are shown in Figure 6G.

### 3.7 PPI, mRNA-miRNA, mRNA-TF, and mRNA-drug interaction networks

**FIGURE 7:**
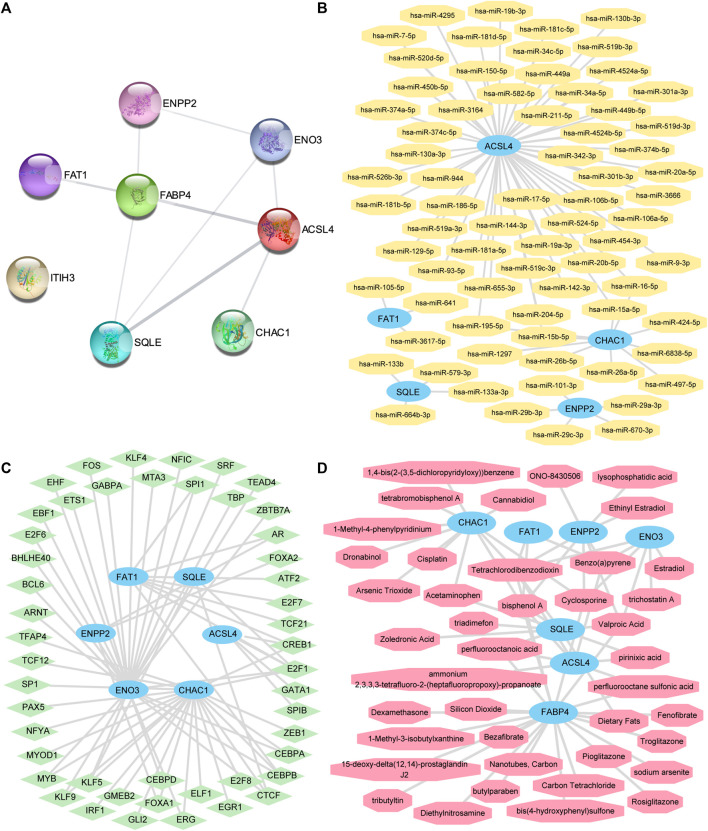
The PPI network, mRNA-miRNA, mRNA-TF, and mRNA-drug interaction networks were constructed. **(A)** The PPI networks of the FRDEGs. **(B)** mRNA-miRNA, **(C)** mRNA-TF, and **(D)** mRNA-drug interaction networks of key genes. The sky-blue oval block in the mRNA-miRNA interaction network **(B)** is mRNA; the orange octagonal blocks represent the miRNAs. The sky-blue oval blocks in the mRNA-TF interaction network **(C)** represent the mRNAs; the light-green diamond blocks represent the TFs. The sky-blue oval blocks in the mRNA-drug interaction network **(D)** represent the mRNAs and the light-red hexagonal blocks represent the specific molecular compounds (drugs). FRDEGs: ferroptosis-related differentially expressed genes; PPI network: protein–protein interaction network; TF: transcription factor.

We used the STRING database for eight FRDEGs in the FRDEG diagnostic model (*ACSL4*, *CHAC1*, *ENO3*, *ENPP2*, *FABP4*, *FAT1*, *ITIH3*, and *SQLE*) for the PPI analysis (minimum required interaction score: medium confidence [0.150]). Eight FRDEG PPI networks were constructed. We used Cytoscape software to visualize the interaction relationships (Figure 7A), with FRDEGs associated with other nodes in the PPI network identified as key genes in MAFLD (hub genes). Seven hub genes were obtained: *ACSL4*, *CHAC1*, *ENO3*, *ENPP2*, *FABP4*, *FAT1*, and *SQLE*.

We predicted that miRNAs interact with these seven key genes using the miRDB database and the mRNA-miRNA data from the ENCORI database. Next, we visualized the mRNA-miRNA interaction network using Cytoscape software (Figure 7B). Our mRNA-miRNA interaction network comprised five hub genes (FRDEGs: *ACSL4*, *CHAC1*, *ENPP2*, *FAT1*, and *SQLE*) and 72 miRNA molecules to form 76 mRNA-miRNA interaction pair relationships. Specific mRNA-miRNA interaction relationships are presented in Supplementary Table S5.

We searched for hub genes binding to TFs through the CHIPBase (version 2.0) and hTFtarget databases. After the interaction relationship was downloaded, the interaction relationship data for six genes (*ACSL4*, *CHAC1*, *ENO3*, *ENPP2*, *FAT1*, and *SQLE*) and 48 TFs were obtained and visualized through the Cytoscape software (Figure 7C). The hub gene *ENO3* had the most interaction relationships (26 pairs) with TF in the mRNA-TF interaction network. Specific mRNA-TF interaction relationships are presented in Supplementary Table S6.

The CTD was used to identify potential drugs or molecular compounds for seven hub genes (*ACSL4*, *CHAC1*, *ENO3*, *ENPP2*, *FABP4*, *FAT1*, and *SQLE*) based on the mRNA-drug interaction network. We identified 41 potential drugs or molecular compounds corresponding to these seven hub genes (Figure 7D). Among them, we found 23 drugs or molecular compounds that target *FABP4*. Specific mRNA-drug interaction relationships are presented in Supplementary Table S7.

### 3.8 Identification of the disease subtypes associated with MAFLD

**FIGURE 8:**
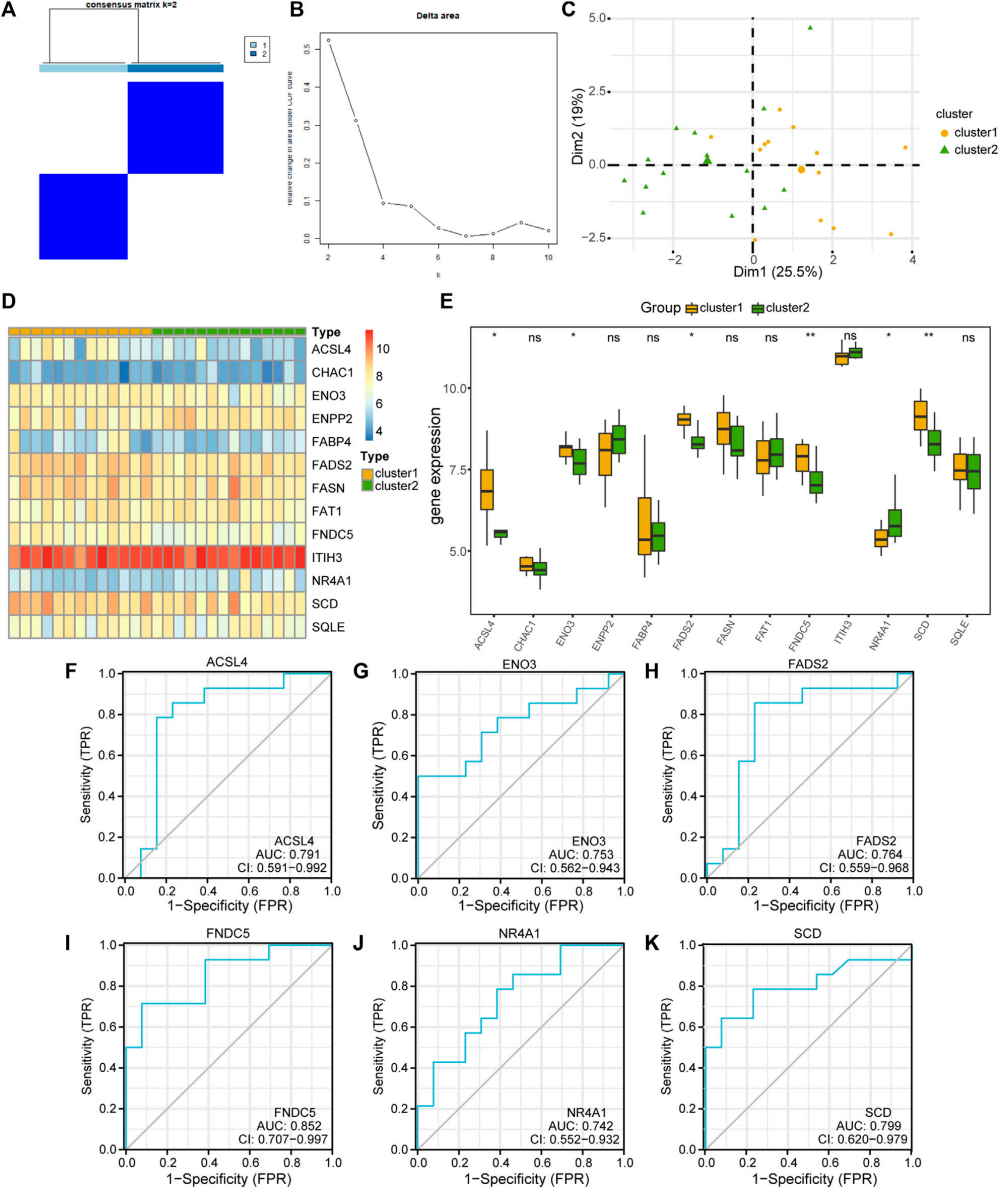
Building the related subtypes of MAFLD. **(A)** Diagram of the consistent clustering results for MAFLD diseases. **(B)** Delta plot of the different numbers of types in the consensus cluster. **(C)** PCA of the two MAFLD subtypes (cluster1 and cluster2). **(D)** A complex numerical heatmap of FRDEGs in different disease subtypes. The color is darker represents the expression of genes is higher. **(E)** Group comparison plots of FRDEGs in different disease subtypes of MAFLD. ns, *p* ≥ 0.05, not significant; *p < 0.05, significant; **p < 0.01, highly significant; and ***p < 0.001, highly significant. **(F–K)** The ROC curves of FRDEGs in the different disease subtypes of MAFLD. **(F)** *ACSL4*, **(G)** *ENO3*, **(H)** *FADS2*, **(I)** *FNDC5*, **(J)** *NR4A1*, and **(K)** *SCD*. In the PCA plots, complex numerical heatmaps, and group comparison plots, the orange and light-green colors indicate cluster1 and cluster2, respectively. MAFLD: metabolic dysfunction-associated fatty liver disease; PCA: principal component analysis; FRDEGs: ferroptosis-related differentially expressed genes; ROC: receiver operating characteristic curve.

To explore the differences in FRDEG expression in the MAFLD group, we used the “ConsensusClusterPlus” package in the R software based on 13 FRDEGs (*ACSL4*, *CHAC1*, *ENO3*, *ENPP2*, *FABP4*, *FADS2*, *FASN*, *FAT1*, *FNDC5*, *ITIH3*, *NR4A1*, *SCD*, and *SQLE*). We used consensus clustering methods to identify the disease subtypes associated with MAFLD. Two MAFLD subtypes (cluster1 and cluster2) were identified (Figures 8A, B). MAFLD subtype 1 (cluster1) contained 13 samples, and MAFLD subtype 2 (cluster2) contained 14 samples. The results of the PCA clustering showed significant differences between the two subtypes of the disease (Figure 8C). We used the R package pheatmap to draw a heatmap showing the differential expression of 13 FRDEGs in the two disease subtypes in the MAFLD dataset (Figure 8D).

Next, we used the Mann–Whitney U test (Wilcoxon rank sum test) to analyze the correlations between the expression levels of the 13 FRDEGs in the MAFLD dataset and the two MAFLD subtypes (cluster1 and cluster2). We displayed the correlation analysis results as group comparison plots (Figure 8E). According to Figure 8E, the expression levels of the FRDEGs (*CHAC1*, *ENPP2*, *FABP4*, *FASN*, *FAT1*, *ITIH3*, and *SQLE*) did not differ significantly between the different MAFLD subtypes (*p* ≥ 0.05), whereas those of *ACSL4*, *ENO3*, *FADS2*, *FNDC5*, *NR4A1*, and *SCD* differed significantly between the different MAFLD subtypes (*p* < 0.05). Subsequently, we plotted the ROC curves of six FRDEGs (*ACSL4*, *ENO3*, *FADS2*, *FNDC5*, *NR4A1*, and *SCD*) in the two disease subtypes of the MAFLD dataset (Figures 8F–K). As shown in Figure 8, the expression levels of *ACSL4* (AUC = 0.791, Figure 8F), *ENO3* (AUC = 0.753, Figure 8G), *FADS2* (AUC = 0.764, Figure 8H), *FNDC5* (AUC = 0.852, Figure 8I), *NR4A1* (AUC = 0.742, Figure 8J), and *SCD* (AUC = 0.799, Figure 8K) in the MAFLD subtypes were somewhat correlated with their grouping in either of the two MAFLD subtypes (cluster1 and cluster2).

### 3.9 Analysis of the differential immune characteristics between the two MAFLD subtypes

**FIGURE 9:**
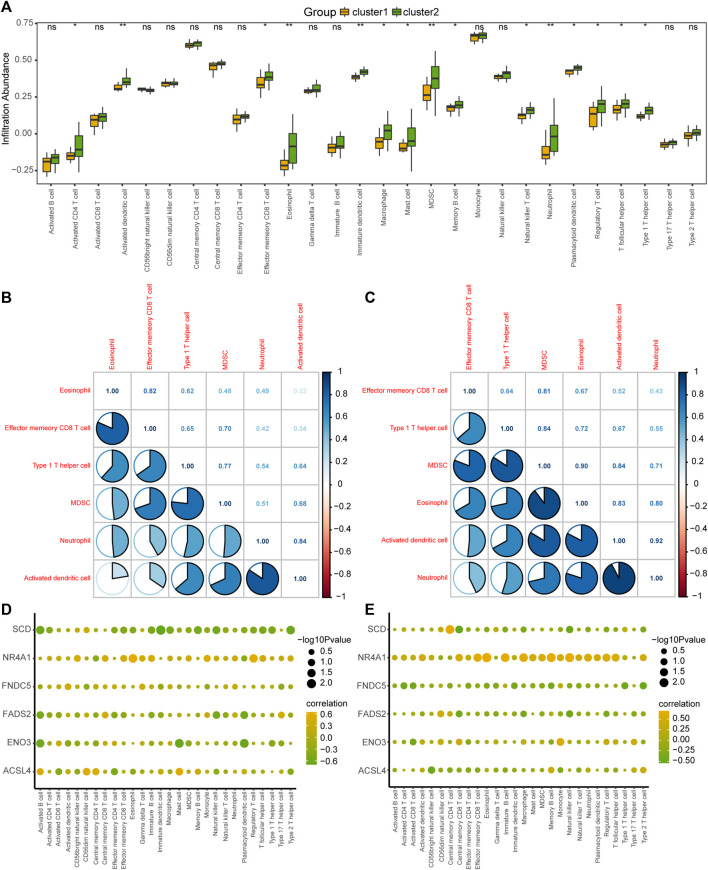
Analysis of ssGSEA immune infiltration between the two MAFLD subtypes. **(A)** Group comparison of ssGSEA immune infiltration analysis results between two MAFLD subtypes. **(B, C)** Results of the correlation analysis of the abundance of infiltrating immune cells in the MAFLD subtypes cluster1 **(B)** and cluster2 **(C)** are shown. **(D, E)** Heatmap of the correlation between immune cells and FRDEGs in the MAFLD subtypes cluster1 **(D)** and cluster2 **(E)**. ns, *p* ≥ 0.05, not significant; *p < 0.05, significant; **p < 0.01, highly significant; and ***p < 0.001, very highly significant. MAFLD: metabolic dysfunction-associated fatty liver disease; ssGSEA: single-sample gene set enrichment analysis; FRDEGs: ferroptosis-related differentially expressed genes.

To explore the difference in immune infiltration between the different MAFLD subtypes (cluster1 and cluster2), we calculated the infiltration abundance of 28 immune cells in the samples of MAFLD subtypes in the MAFLD dataset using the ssGSEA algorithm, analyzed the infiltration abundance using the Mann–Whitney U test, and displayed the results as a group comparison plot (Figure 9A). The results showed that the infiltration abundance of 15 immune cells varied significantly between the MAFLD subtypes in the MAFLD dataset (*p* < 0.05). These immune cell types were activated CD4^+^ T cells, activated dendritic cells, effector memory CD8^+^ T cells, eosinophils, immature dendritic cells, macrophages, mast cells, myeloid-derived suppressor cells (MDSCs), memory B cells, natural killer T cells, neutrophils, plasmacytoid dendritic cells, regulatory T cells, T follicular helper cells, and Type 1 T helper cells. Among them, the infiltration abundance of activated dendritic cells, eosinophils, immature dendritic cells, MDSCs, and neutrophils differed between the MAFLD subtypes, with substantial significance (*p* < 0.01).

We calculated the correlation between the infiltration abundance of 28 immune cells in the cluster1 and cluster2 samples and six selected immune cells (eosinophils, effector, and memory CD8^+^ T cells, Type 1 T helper cells, MDSCs, neutrophils, and activated dendritic cells), with high correlations (Figures 9B, C). The results for the cluster1 and cluster2 subtypes are shown in Figures 9B, C, respectively. The greatest correlation was observed between the infiltration abundance of neutrophils and activated dendritic cells (Figures 9B, C).

We also calculated the correlation between the infiltration abundance of 28 immune cells and the expression of six FRDEGs (*ACSL4*, *ENO3*, *FADS2*, *FNDC5*, *NR4A1*, and *SCD*) in the patient samples of cluster1 (Figure 9D) and cluster2 (Figure 9E). The results showed a significant correlation between immune cell abundance and the expression of the six FRDEGs in both subtypes. Among the FRDEGs, *NR4A1* expression showed a significant positive correlation with the infiltration abundance of most immune cells in both MAFLD subtypes.

**FIGURE 10:**
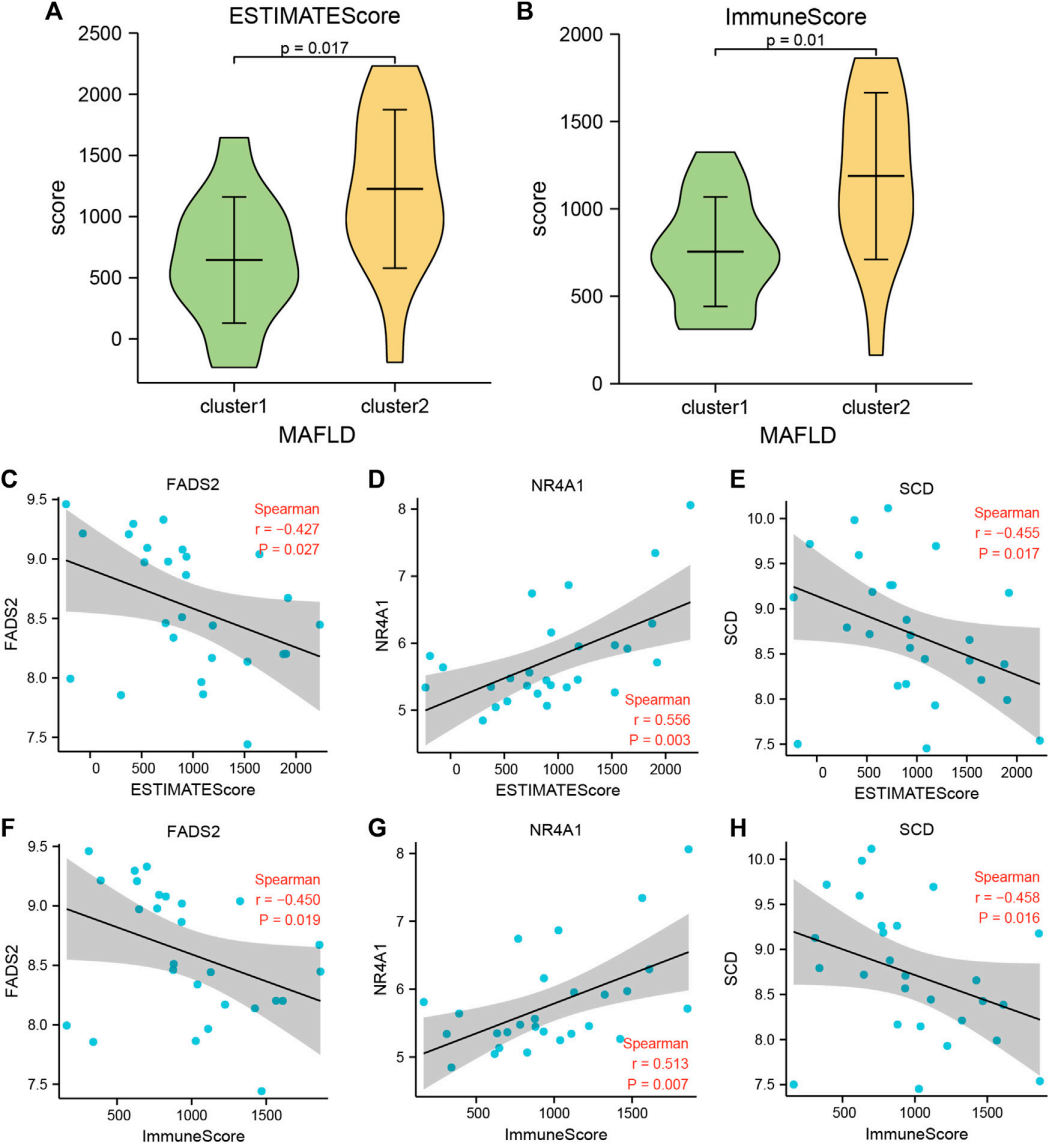
Immune scores between the two MAFLD disease subtypes (estimate). **(A, B)** The immune score results are shown between the two MAFLD subtypes. **(A)** ESTIMATE, **(B)** Immune. **(C–E)** Scatter plot of the correlation between the ESTIMATE Score of the two MAFLD subtypes and the FRDEGs *FNDC5* **(C)**, *NR4A1* **(D)**, and *SCD* **(E)**. **(F–H)** Scatter plots of correlation between the Immune Scores of the two MAFLD subtypes and the FRDEGs *FNDC5* **(F)**, *NR4A1* **(G)**, and *SCD* **(H)**. If the correlation coefficient (r) value in the correlation scatter plot is positive, the two variables may show a positive correlation; conversely, if the r value is negative, the two variables may show a negative correlation. Furthermore, absolute r values above 0.8, between 0.5 and 0.8, between 0.3 and 0.5, and <0.3 indicate a strong, moderate, general, and weak or no correlation, respectively. The horizontal axis of the correlation scatter plot represents the immune score of the samples of the MAFLD subtypes cluster1 and cluster2, and the vertical axis represents the expression levels of the FRDEGs. *p* ≥ 0.05, not significant; *p* < 0.05, significant; *p* < 0.01, highly significant; *p* < 0.001, very highly significant. MAFLD: metabolic dysfunction-associated fatty liver disease.

The ESTIMATE package used the unique transcriptional profiles of cancer samples to infer the content of tumor cells and different infiltrating normal cells, mainly through RNA-seq data, to calculate the immune and mechanistic score. We then evaluated the purity of the tumor. Subsequently, we assessed the collated MAFLD dataset expression profile data using the R ESTIMATE package and obtained the immune and matrix scores for samples from the NAFLD subtypes, cluster1, and cluster2, in the MAFLD dataset. After processing the results, we obtained the ESTIMATE, Immune, and Stromal scores of these samples. We showed the score results by group comparison diagram (Figures 10A, B, Supplementary Figure S1A), the ESTIMATE (Figure 10A) and Immune (Figure 10B) scores differed significantly between the MAFLD subtypes (*p* < 0.05), whereas the Stromal score (Figure 10C) did not differ significantly between subtypes (*p* > 0.05).

We analyzed the correlation between the ESTIMATE and Immune Scores of the two MAFLD disease subtype groups and the expression levels of six FRDEGs (*ACSL4*, *ENO3*, *FADS2*, *FNDC5*, *NR4A1*, and *SCD*) and displayed the specific analysis results using a correlation scatter plot (Figures 10C–H; Supplementary Figure S2B-G). The MAFLD disease subtype, cluster1, cluster2, and the grouped sample ESTIMATE and Immune Scores compared with the expression levels of *FADS2*, *NR4A1*, and *SCD* were statistically significant (*p <* 0.05). We discovered negative correlation and moderate positive correlation between the expression levels of *SCD* (−0.3 > r > −0.5) and the *NR4A1* (0.5 < r < 0.8) and ESTIMATE and Immune Scores, respectively. However, the expression levels of *ACSL4*, *ENO3*, and *FNDC5 were* negatively correlated with the ESTIMATE and Immune Scores, but this did not reach statistical significance (r < 0, *p* > 0.05).

### 3.10 Differential analysis of FRDEG expression in clinical subgroups

**FIGURE 11:**
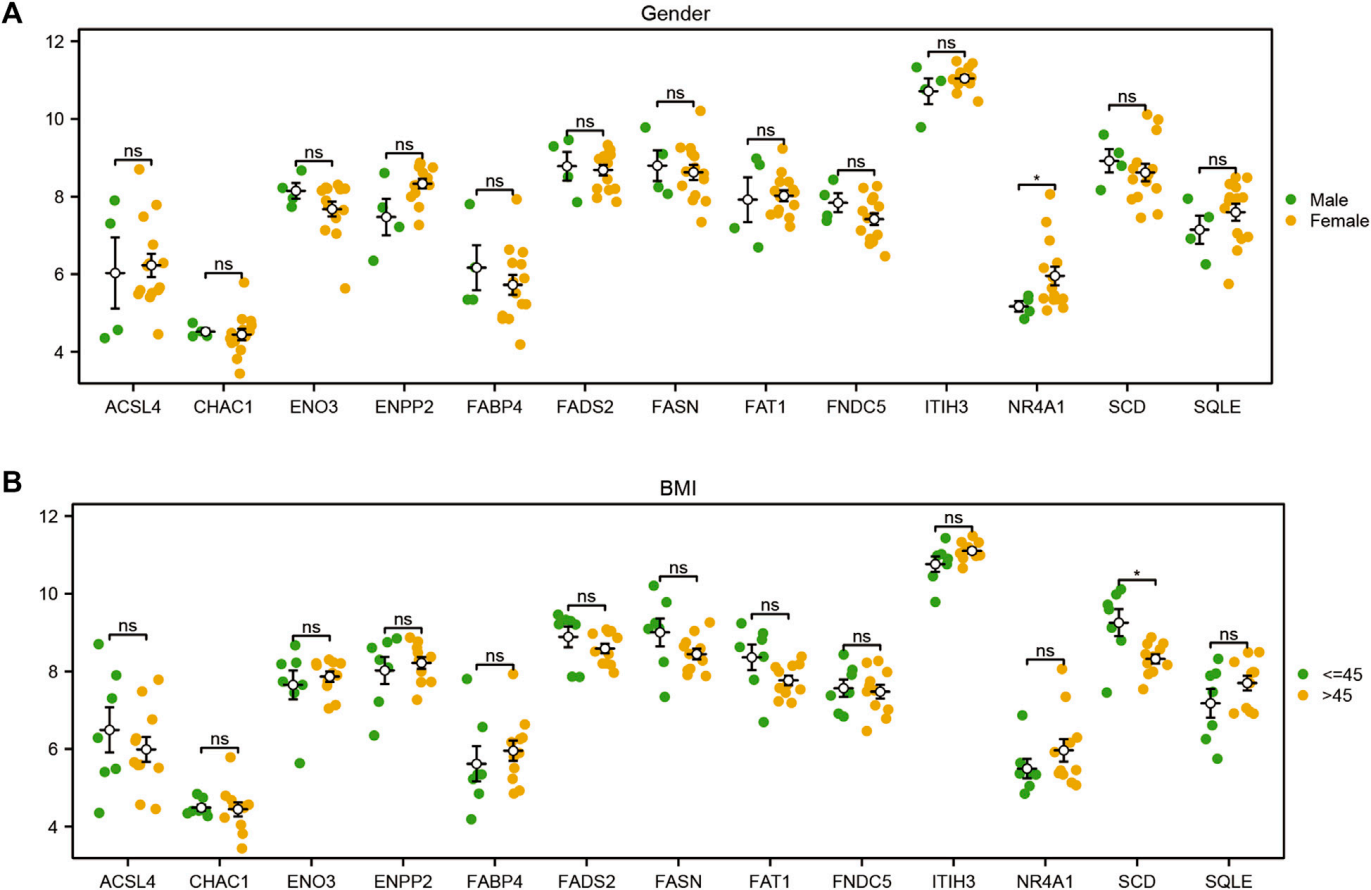
Differential analysis of FRDEG expression in clinical subgroups. Group comparison diagram of the analyses of the differences in FRDEG expression with regard to different clinical characteristics (sex, BMI) in the MAFLD dataset. ns, *p* ≥ 0.05, not significant; *p < 0.05, significant; **p < 0.01, highly significant; ***p < 0.001, very highly significant. FRDEGs: ferroptosis-related differentially expressed genes. The validation of the expression of candidate genes in the cell MAFLD model.

To explore the difference in the expression levels of the 13 FRDEGs (*ACSL4*, *CHAC1*, *ENO3*, *ENPP2*, *FABP4*, *FADS2*, *FAT1*, *FNDC5*, *ITIH3*, *NR4A1*, *SCD*, and *SQLE*) in the GSE48452 of the MAFLD dataset, we first compiled the clinical information from the 32 samples of the GSE48452 dataset and drew the clinical information table (Supplementary Table S8). We combined specific clinical characteristic-associated grouping information (sex, age, BMI, leptin, adiponectin) and analyzed the expression levels of the 13 FRDEGs in GSE48452 samples of the MAFLD dataset and the difference in expression levels between different groups. We showed the results of the expression difference analysis through the group comparison diagram (Figure 11; Supplementary Figure S2). According to Figure 11, only the expression level of *NR4A1* was statistically significant (symbol * equivalent to *p <* 0.05) among the 13 FRDEGs, with different clinical information sex groups (male/female) of the MAFLD samples, and only *SCD* was statistically significant between different clinical information BMI groups (≤45/> 45) (symbol * equivalent to *p <* 0.05). However, in different groups of clinical information (Supplementary Figure S2), including the age (≤45/> 45), leptin (≤30/> 30), and adiponectin groups (≤6/> 6), no genes had statistically significant expression (symbol ns equivalent to *p >* 0.05).

### 3.11 Validation of the expression of candidate genes in the cell MAFLD model

**FIGURE 12:**
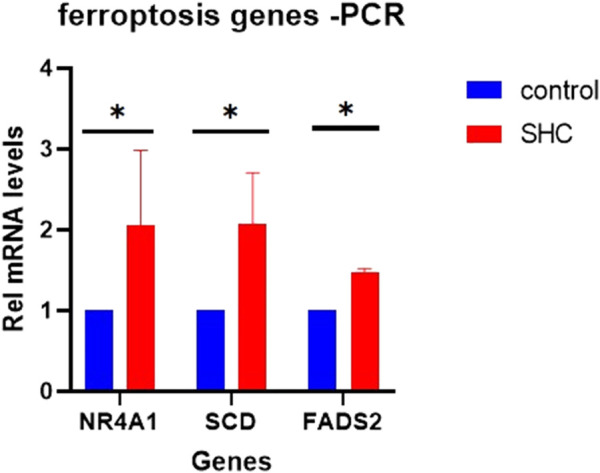
Expression of candidate genes in the cell MAFLD model. **(A)** The expression of *NR4A1*, *FADS2*, and *SCD* in a cell model of MAFLD. *p < 0.05, statistically significant. MAFLD: metabolic dysfunction-associated fatty liver disease.

*NR4A1*, *FADS2*, and *SCD* were important in ferroptosis-related immune infiltration of MAFLD. We validated the above genes in a cell model of MAFLD using RT-qPCR. The PCR results showed that compared with the Control group, the expressions of these three genes were higher in the SHC groups than in the Control groups, which were statistically significant (Figure 12).

## 4 Discussion

**FIGURE 13:**
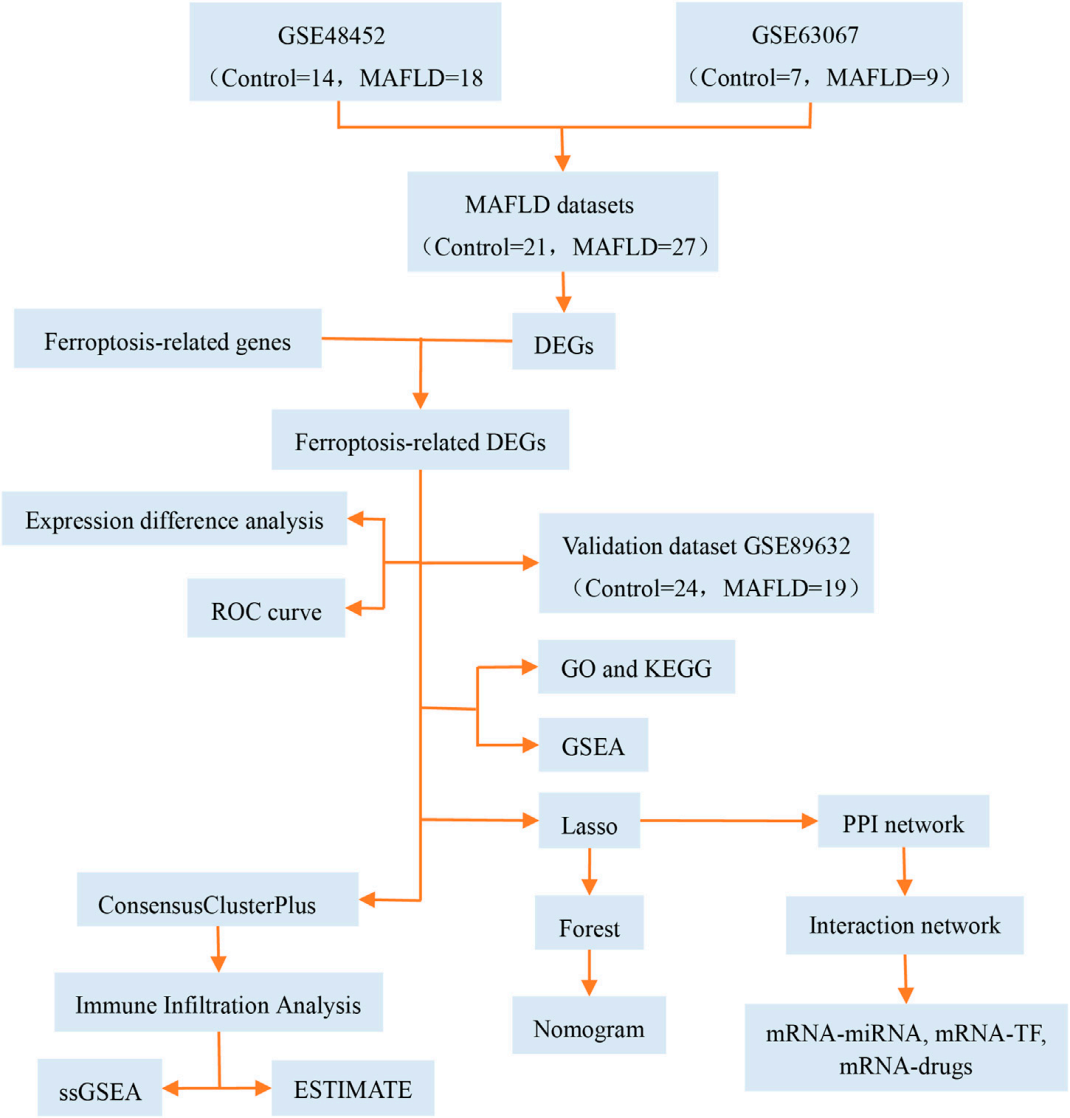
Technical roadmap. GO: Gene Ontology; GSEA: gene set enrichment analysis; KEGG: Kyoto Encyclopedia of Genes and Genomes; LASSO: least absolute shrinkage and selection operator; MAFLD: metabolic dysfunction-associated fatty liver disease; DEGs: differentially expressed genes; ROC: receiver operating characteristic curve; ssGSEA: single-sample gene set enrichment analysis; TF: transcription factors.

MAFLD has become a major public health concern (Younossi et al., 2016) that affects one-third of adults in western nations and is more prevalent in Asian countries (Li et al., 2019). Limited pharmacological therapy is available for patients with MAFLD. Ferroptosis was recently identified as a nonapoptotic form of cell death (Dixon et al., 2012). Modulation of ferroptosis may have therapeutic potential in some ferroptosis-related diseases. However, the pathological process of ferroptosis in MAFLD remains unclear. Previous reports have stated that the pathological process of ferroptosis in MAFLD could be directly induced by iron overload or ferroptosis-related pathways involving selenium and selenoproteins (Jia et al., 2021; Wu et al., 2021; Zhou et al., 2022). In this study, we focused on the role of ferroptosis in the development of MAFLD. We analyzed ferroptosis-related genes using bioinformatics methods, constructed diagnostic and prognostic models, and explored the immunologic characteristics of MAFLD (Figure 13), and provided clues in the search for novel therapeutic targets for MAFLD, particularly for therapeutic strategies targeting the ferritin and ferroptosis pathways, which might have a positive impact on improving the health of the MAFLD patients.

Our findings revealed that ferroptosis-related genes were closely related to the DEGs of MAFLD. A total of 13 MAFLD FRDEGs were obtained: *ACSL4*, *CHAC1*, *ENO3*, *ENPP2*, *FABP4*, *FADS2*, *FASN*, *FAT1*, *FNDC5*, *ITIH3*, *NR4A1*, *SCD*, and *SQLE*. Among them, *ENO3*, *FABP4*, *FADS2*, *FAT1*, and *ITIH3* were expressed at different levels between the different groups in the MAFLD dataset, with a high degree of statistical significance. Eight FRDEGs (*ACSL4*, *CHAC1*, *ENO3*, *ENPP2*, *FABP4*, *FAT1*, *ITIH3*, and *SQLE*) were used to construct diagnostic models. The results showed that the FRDEG diagnostic model could effectively predict the occurrence of steatohepatitis in patients with MAFLD (AUC = 0.922, Figure 6D). The diagnostic effectiveness of FRDEGs in MAFLD has not previously been investigated, despite being widely acknowledged as diagnostic and/or prognostic indicators in various tumor types, such as hepatocellular carcinoma (Liang et al., 2020), breast cancer (Lu et al., 2022), pancreatic adenocarcinoma (Yang et al., 2022), cholangiocarcinoma (Wang et al., 2022), and lung adenocarcinoma (Li F. et al., 2021). To our knowledge, this study is the first to investigate the diagnostic use of FRDEGs in MAFLD. Eight FRDEGs were analyzed using multivariate Cox regression analysis, and a diagnostic model was constructed. Furthermore, a nomogram was successfully constructed to predict the risk of steatohepatitis. Finally, we used a DCA to evaluate the clinical utility of the constructed Cox regression model (Figure 6G). Therefore, the prognostic signature could determine the risk of steatohepatitis in patients with MAFLD and contribute to therapeutic guidelines.

The results of GO analysis, KEGG pathway analysis, and GSEA of the 13 FRDEGs indicated that the pathways were related to fatty acid biosynthesis and metabolism, PPAR signaling, and cholesterol biosynthesis. This is consistent with previous studies that confirmed ferroptosis-related gene participation in MAFLD progression through these signaling pathways (Lu et al., 2021; Yao et al., 2023). We used the STRING database for PPI analysis, and seven hub genes were identified: *ACSL4*, *CHAC1*, *ENO3*, *ENPP2*, *FABP4*, *FAT1*, and *SQLE*. An mRNA-miRNA interaction network comprising five hub genes (FRDEGs: *ACSL4*, *CHAC1*, *ENPP2*, *FAT1*, and *SQLE*) and 72 miRNA molecules, forming 76 mRNA-miRNA interaction pair relationships, were constructed. Future research can explore this mRNA-miRNA interaction network further, like investigating miRNA alteration or DNA methylation (Anuraga et al., 2021; Modhukur et al., 2018; Xing et al., 2021). Furthermore, we constructed mRNA-TF interaction networks and discovered that the key gene *ENO3* had the most interactions with TFs, forming 26 mRNA-TF interaction pair relationships. A study has shown that *ENO3* promoted the progression of NASH by negatively regulating ferroptosis (Lu et al., 2021). A recent review (Jia et al., 2021) summarized ferroptosis-related drugs, whose inhibition targets were System xc-, *GPX4*, PUFAs, HMG-CoA reductase, *ACSL4*, HO-1, and *NRF2*. Through the CTD, our study identified 41 potential drugs or molecular compounds corresponding to seven key genes, including *ACSL4*, which represented potential drugs or molecular compounds. A study (Tsurusaki et al., 2019) used rosiglitazone, an *ACSL4* inhibitor, to investigate ferroptosis in patients with MAFLD. We aim to conduct a more in-depth study of this gene.

Innate immunity is highly involved in MAFLD development. Increasing evidence supports the significant immunological contribution to the pathology of steatohepatitis (Carranza-Trejo et al., 2021; Nati et al., 2022), and ferroptosis-related immune infiltration has also been inferred. According to Linkermann et al., cells undergoing ferroptosis emit damage-associated chemical patterns that cause inflammation and activate the innate immune system (Tang et al., 2011). Furthermore, Tsurusaki et al. demonstrated that hepatic ferroptosis is crucial for triggering inflammation in people with MAFLD (Tsurusaki et al., 2019).

In this study, two subtypes (cluster1 and cluster2) of MAFLD were identified. The results showed that the infiltration abundance of 15 immune cells differed significantly between the two MAFLD subtypes (*p* < 0.05), and the infiltration abundance of these immune cells was higher in cluster2 than in cluster1 (Figure 10A). This finding suggests that cluster2 may represent an “immunoactive” type, whereas cluster1 was the “immunosuppressive” type, in which neutrophils and activated dendritic cells (ADCs) appeared to be the two most abundant of the 15 immune cell types (Figures 9B, C). According to previous research, the neutrophil-to-lymphocyte ratio is significantly and independently related to advanced inflammation and fibrosis and may represent a valid diagnostic biomarker for steatohepatitis and terminal fibrosis in patients with MAFLD (Alkhouri et al., 2012; Khoury et al., 2019). Infiltrating neutrophils in the liver release cytokines, which can alter the progression of steatohepatitis (Wang et al., 2021); therefore, neutrophils are strongly associated with the development of fibrosis in patients with MAFLD. ADCs connect the innate and adaptive immune responses by internalizing antigens and transporting them to local lymph nodes (Jenne and Kubes, 2013; Schuster et al., 2018). The function of ADCs in MAFLD remains unclear and controversial. According to Henning et al., ADC depletion worsens hepatic fibrosis and inflammation, indicating that ADCs slow the progression of steatohepatitis (Henning et al., 2013). In contrast, Connolly et al. suggested that ADCs promote the progression of liver fibrosis and inflammation in NASH (Connolly et al., 2009); this is consistent with our findings.

We calculated the correlation between the infiltration abundance of 28 immune cells and the expression of six FRDEGs (*ACSL4*, *ENO3*, *FADS2*, *FNDC5*, *NR4A1*, and *SCD*) in patient samples of cluster1 (Figure 9D) and cluster2 (Figure 9E) and showed a significant correlation between immune cell content and the expression of three FRDEGs (*NR4A1*, *FADS2*, and *SCD*). We validated the expression of the three genes in the MAFLD cell model. The results showed that the expression of the genes in the steatohepatitis group was significantly higher than that in the Control group, which matched the bioinformatics analysis. *NR4A1* is a nuclear receptor of the NR4A family, which primarily acts as a transcription factor to regulate the expression of multiple genes. In MAFLD development, hyperactivated *NR4A1* preferentially drives DNA-PKcs/p53 signaling, resulting in mitochondrial dysfunction (Zhou et al., 2018). *FADS2* is a crucial enzyme involved in the metabolism of n-3 and n-6 polyunsaturated fatty acids, which enable alpha-linolenic acid and linoleic acid to generate long-chain polyunsaturated fatty acids, contributing to MAFLD development. Numerous investigations have indicated that increased *FADS2* expression may play a role in NAFLD pathophysiology (Arendt et al., 2015; Chen et al., 2017; Walle et al., 2019). *SCD* is a key gene involved in lipid metabolism and ferroptosis. Recent research has outlined how *SCD* influences cancer progression through its effects on lipid metabolism and cell proliferation, migration, invasion, and metastasis (Wohlhieter et al., 2020). These studies are consistent with our findings. Therefore, immunological mechanisms play a key role in MAFLD pathogenesis. Future, we aim to target the above genes to slow down the development of MAFLD through immunotherapy.

This study had a few limitations. First, due to the retrospective study design and the use of publicly available data, we could not include additional demographic and clinical variables, such as illness progress, complications, and individual treatment, for more extensive longitudinal analyses. Second, the public data for the current study MAFLD were all obtained from NAFLD patients, which leaded to the analysis can not fully explain the pathogenesis of MAFLD. Third, our list of ferroptosis-related genes was not complete because it was obtained from the constantly updated FerrDb. More research is needed to evaluate the mechanisms of these ferroptosis-related genes in MAFLD, like using Cancer Cell Line Encyclopedia (CCLE) database (Barretina et al., 2012; Kao et al., 2021; Li et al., 2021b) or Proteinatlas or cBioportal (Cerami et al., 2012; Lamb et al., 2006; Wang et al., 2020)to validate the results and investigate their role in fibrosis progression to cancer stage.

## 5 Conclusion

Summarily, we identified eight hub genes (*ACSL4*, *CHAC1*, *ENO3*, *ENPP2*, *FABP4*, *FAT1*, *ITIH3*, and *SQLE*) that are potential ferroptosis-related biomarkers for disease diagnosis and prognosis. The expression of three hub genes (*FADS2*, *NR4A1*, and *SCD*) correlated with the infiltration abundance of most immune cells in the two MAFLD subtypes. Therefore, a ferroptosis-related gene signature was successfully constructed for the diagnosis and prognosis of steatohepatitis in MAFLD and can promote the establishment of novel therapeutic approaches and directly tailored therapy, including immunotherapy.

## Data Availability

The datasets presented in this study can be found in online repositories. The names of the repository/repositories and accession number(s) can be found in the article/Supplementary Material.
