# Supplementary material for: Use of a ferroptosis-related gene signature to construct diagnostic and prognostic models for assessing immune infiltration in metabolic dysfunction-associated fatty liver disease
Source: Front Cell Dev Biol. 2023 Oct 19;11:1199846. doi: 10.3389/fcell.2023.1199846 (PMC10622674; doi:10.3389/fcell.2023.1199846)
Supplement: Supplementary file 1 [file Table8.docx]

#### Table S8. Patient Characteristics of MAFLD patients in the GSE48452 datasets.

| Characteristics | Control | MAFLD | P value |
| --- | --- | --- | --- |
| n | 14 | 18 |  |
| gender, n (%) |  |  | 0.453 |
| Male | 5 (15.6%) | 4 (12.5%) |  |
| Female | 9 (28.1%) | 14 (43.8%) |  |
| Age, mean ± sd | 51.828 ± 17.677 | 45.476 ± 8.9258 | 0.235 |
| BMI, mean ± sd | 25.145 ± 3.8227 | 45.972 ± 12.961 | < 0.001 |
| Leptin, mean ± sd | 6.8404 ± 6.4132 | 35.66 ± 24.35 | < 0.001 |
| Adiponectin, median (IQR) | 10.697 (6.0992, 12.31) | 6.404 (5.5424, 7.8465) | 0.038 |

MAFLD: Metabolic dysfunction-associated fatty liver disease

BMI:Body Mass Index;

IQR:Interquartile range.
