# Supplementary figures and images for: Use of a ferroptosis-related gene signature to construct diagnostic and prognostic models for assessing immune infiltration in metabolic dysfunction-associated fatty liver disease
Source: Front Cell Dev Biol. 2023 Oct 19;11:1199846. doi: 10.3389/fcell.2023.1199846 (PMC10622674; doi:10.3389/fcell.2023.1199846)

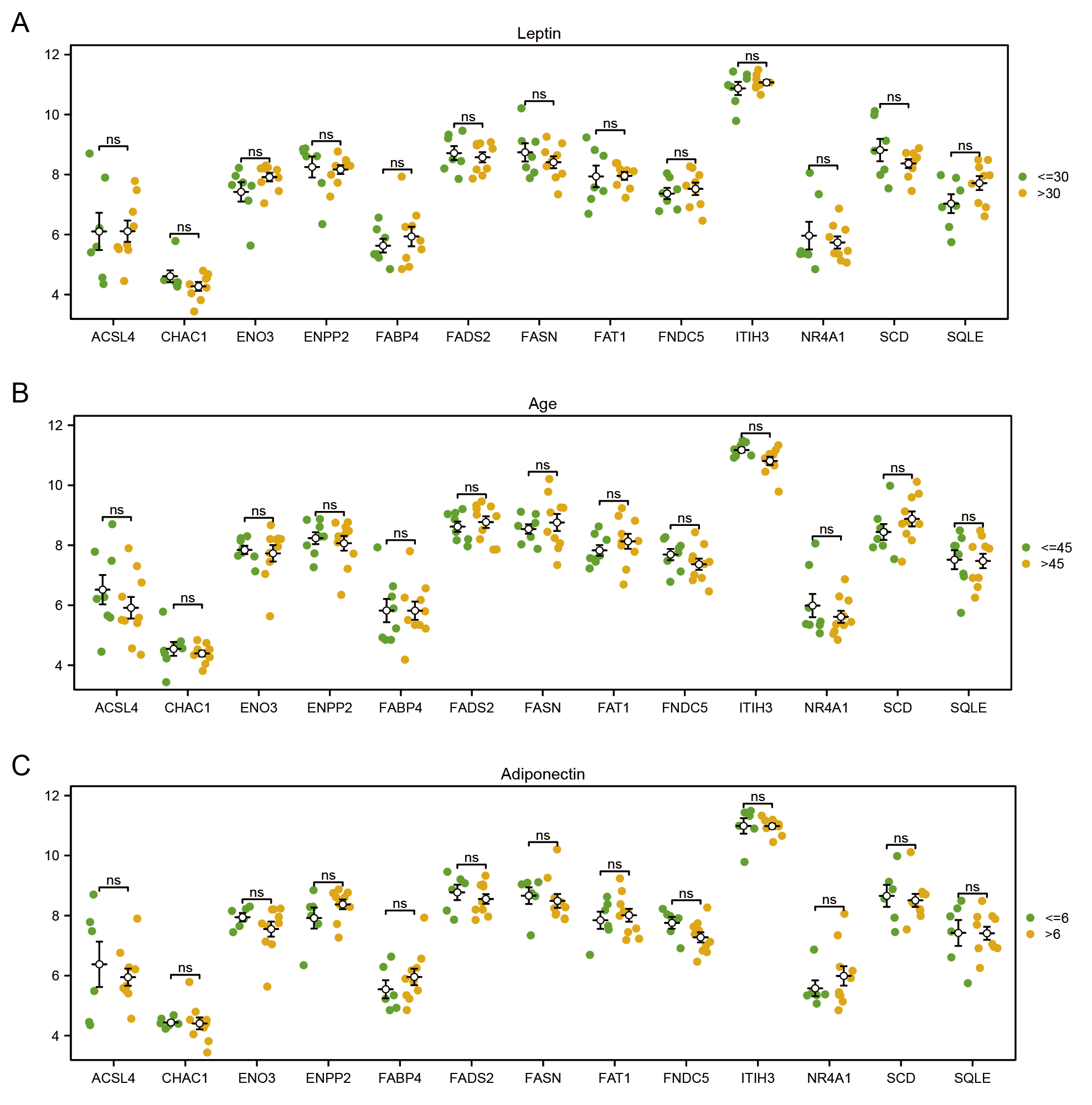

Supplement: Supplementary file 2 [file Image2.tif]

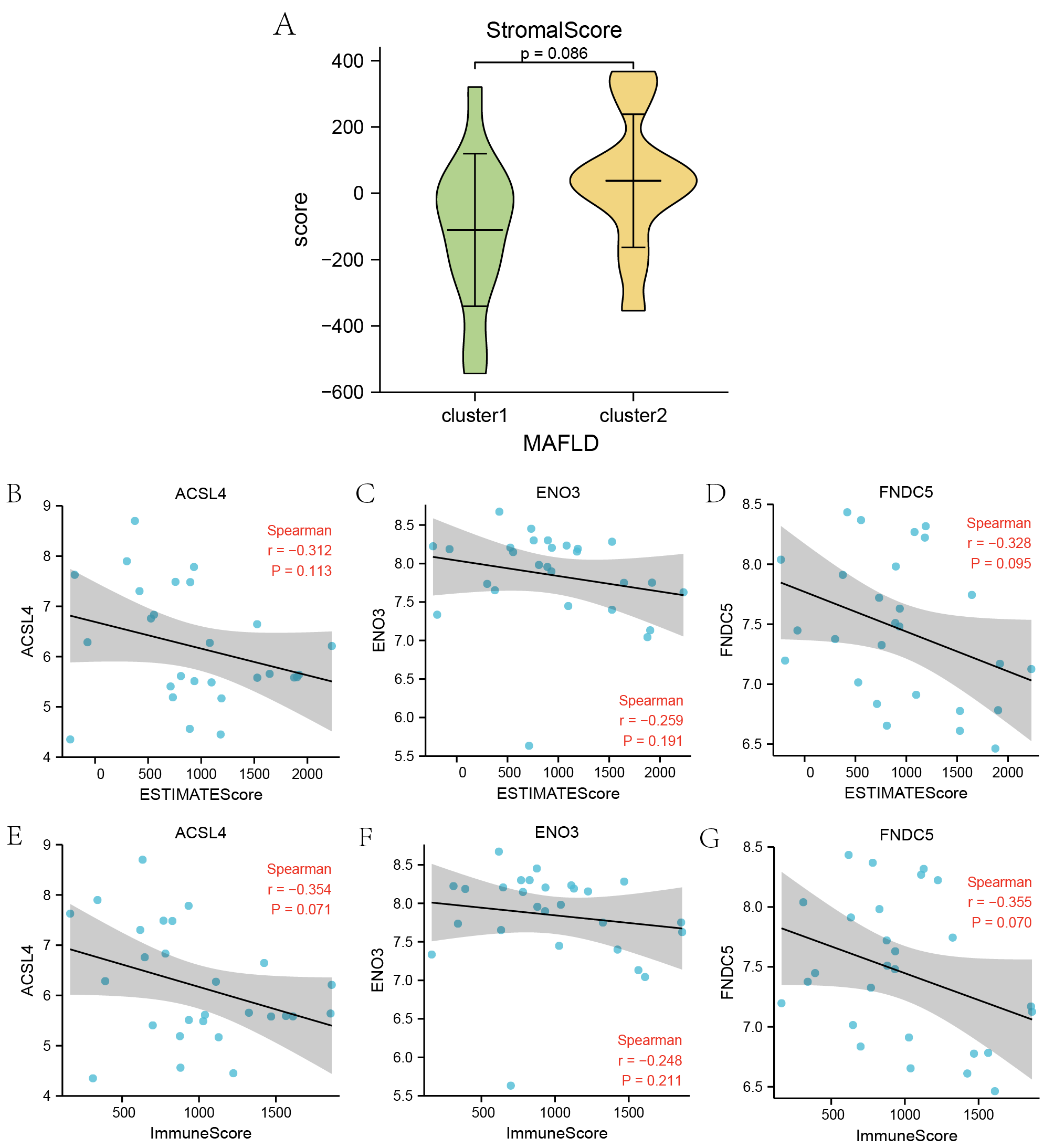

Supplement: Supplementary file 4 [file Image1.tif]
