# Supplementary material for: Use of a ferroptosis-related gene signature to construct diagnostic and prognostic models for assessing immune infiltration in metabolic dysfunction-associated fatty liver disease
Source: Front Cell Dev Biol. 2023 Oct 19;11:1199846. doi: 10.3389/fcell.2023.1199846 (PMC10622674; doi:10.3389/fcell.2023.1199846)
Supplement: Supplementary file 3 [file Table7.docx]

### Table S7. mRNA-drugs interaction network nodes.

| mRNA |  | drug |  | mRNA |  | drug |
| --- | --- | --- | --- | --- | --- | --- |
| ACSL4 | - | Tetrachlorodibenzodioxin |  | FABP4 | - | Troglitazone |
| ACSL4 | - | bisphenol A |  | FABP4 | - | Dietary Fats |
| ACSL4 | - | pirinixic acid |  | FABP4 | - | bis（4-hydroxyphenyl）sulfone |
| ACSL4 | - | Benzo（a）pyrene |  | FABP4 | - | Bezafibrate |
| ACSL4 | - | perfluorooctane sulfonic acid |  | FABP4 | - | tributyltin |
| ACSL4 | - | perfluorooctanoic acid |  | FABP4 | - | Dexamethasone |
| ACSL4 | - | ammonium 2,3,3,3-tetrafluoro-2-（heptafluoropropoxy）-propanoate |  | FABP4 | - | Tetrachlorodibenzodioxin |
| CHAC1 | - | bisphenol A |  | FABP4 | - | Pioglitazone |
| CHAC1 | - | 1-Methyl-4-phenylpyridinium |  | FABP4 | - | 1-Methyl-3-isobutylxanthine |
| CHAC1 | - | Dronabinol |  | FABP4 | - | butylparaben |
| CHAC1 | - | Cyclosporine |  | FABP4 | - | Carbon Tetrachloride |
| CHAC1 | - | Acetaminophen |  | FABP4 | - | perfluorooctanoic acid |
| CHAC1 | - | Tetrachlorodibenzodioxin |  | FABP4 | - | 15-deoxy-delta（12,14）-prostaglandin J2 |
| CHAC1 | - | Cannabidiol |  | FABP4 | - | Cyclosporine |
| CHAC1 | - | tetrabromobisphenol A |  | FABP4 | - | Diethylnitrosamine |
| CHAC1 | - | 1,4-bis（2-（3,5-dichloropyridyloxy））benzene |  | FABP4 | - | Fenofibrate |
| CHAC1 | - | Arsenic Trioxide |  | FABP4 | - | Nanotubes, Carbon |
| CHAC1 | - | Cisplatin |  | FABP4 | - | perfluorooctane sulfonic acid |
| ENO3 | - | Tetrachlorodibenzodioxin |  | FABP4 | - | Silicon Dioxide |
| ENO3 | - | Benzo（a）pyrene |  | FABP4 | - | sodium arsenite |
| ENO3 | - | Estradiol |  | FABP4 | - | Valproic Acid |
| ENO3 | - | trichostatin A |  | FAT1 | - | Tetrachlorodibenzodioxin |
| ENO3 | - | Valproic Acid |  | FAT1 | - | bisphenol A |
| ENO3 | - | Cyclosporine |  | FAT1 | - | Benzo（a）pyrene |
| ENPP2 | - | Tetrachlorodibenzodioxin |  | SQLE | - | bisphenol A |
| ENPP2 | - | ONO-8430506 |  | SQLE | - | perfluorooctanoic acid |
| ENPP2 | - | bisphenol A |  | SQLE | - | Tetrachlorodibenzodioxin |
| ENPP2 | - | Ethinyl Estradiol |  | SQLE | - | Acetaminophen |
| ENPP2 | - | Benzo（a）pyrene |  | SQLE | - | triadimefon |
| ENPP2 | - | lysophosphatidic acid |  | SQLE | - | Zoledronic Acid |
| ENPP2 | - | Cyclosporine |  | SQLE | - | Dietary Fats |
| ENPP2 | - | Valproic Acid |  | SQLE | - | pirinixic acid |
| FABP4 | - | bisphenol A |  | SQLE | - | Valproic Acid |
| FABP4 | - | Rosiglitazone |  |  |  |  |

“mRNA”and“drug”represent node；“-”represent edge。
