# Supplementary material for: Use of a ferroptosis-related gene signature to construct diagnostic and prognostic models for assessing immune infiltration in metabolic dysfunction-associated fatty liver disease
Source: Front Cell Dev Biol. 2023 Oct 19;11:1199846. doi: 10.3389/fcell.2023.1199846 (PMC10622674; doi:10.3389/fcell.2023.1199846)
Supplement: Supplementary file 5 [file Table2.docx]

Table S2. GO enrichment analysis results of Ferroptosis-related differentially expressed genes.

| Ontology | ID | Description | GeneRatio | BgRatio | pvalue | p.adjust | qvalue |
| --- | --- | --- | --- | --- | --- | --- | --- |
| BP | GO:0051188 | cofactor biosynthetic process | 5/13 | 326/18670 | 1.81e-06 | 2.82e-04 | 1.49e-04 |
| BP | GO:0044272 | sulfur compound biosynthetic process | 4/13 | 192/18670 | 7.21e-06 | 2.82e-04 | 1.49e-04 |
| BP | GO:0009108 | coenzyme biosynthetic process | 4/13 | 261/18670 | 2.42e-05 | 4.45e-04 | 2.35e-04 |
| BP | GO:0072330 | monocarboxylic acid biosynthetic process | 4/13 | 343/18670 | 7.02e-05 | 8.14e-04 | 4.30e-04 |
| CC | GO:0005778 | peroxisomal membrane | 2/13 | 60/19717 | 6.95e-04 | 0.013 | 0.010 |
| CC | GO:0031903 | microbody membrane | 2/13 | 60/19717 | 6.95e-04 | 0.013 | 0.010 |
| CC | GO:0005811 | lipid droplet | 2/13 | 81/19717 | 0.001 | 0.014 | 0.010 |
| CC | GO:0044438 | microbody part | 2/13 | 100/19717 | 0.002 | 0.014 | 0.010 |
| MF | GO:0016829 | lyase activity | 3/13 | 187/17697 | 3.07e-04 | 0.011 | 0.006 |
| MF | GO:0016836 | hydro-lyase activity | 2/13 | 56/17697 | 7.50e-04 | 0.019 | 0.010 |
| MF | GO:0051427 | hormone receptor binding | 2/13 | 185/17697 | 0.008 | 0.064 | 0.034 |
| MF | GO:0050662 | coenzyme binding | 2/13 | 291/17697 | 0.019 | 0.064 | 0.034 |

GO：Gene Ontology；BP：biological process；CC：cellular component；MF：molecular function.
