# Supplementary material for: Use of a ferroptosis-related gene signature to construct diagnostic and prognostic models for assessing immune infiltration in metabolic dysfunction-associated fatty liver disease
Source: Front Cell Dev Biol. 2023 Oct 19;11:1199846. doi: 10.3389/fcell.2023.1199846 (PMC10622674; doi:10.3389/fcell.2023.1199846)
Supplement: Supplementary file 6 [file Table5.docx]

**Table S5. mRNA-miRNA interaction network nodes.**

| mRNA |  | miRNA |  | mRNA |  | miRNA |
| --- | --- | --- | --- | --- | --- | --- |
| ACSL4 | - | hsa-miR-130a-3p |  | ACSL4 | - | hsa-miR-142-3p |
| ACSL4 | - | hsa-miR-301a-3p |  | ACSL4 | - | hsa-miR-34c-5p |
| ACSL4 | - | hsa-miR-130b-3p |  | ACSL4 | - | hsa-miR-450b-5p |
| ACSL4 | - | hsa-miR-454-3p |  | ACSL4 | - | hsa-miR-342-3p |
| ACSL4 | - | hsa-miR-301b-3p |  | ACSL4 | - | hsa-miR-144-3p |
| ACSL4 | - | hsa-miR-4295 |  | ACSL4 | - | hsa-miR-449a |
| ACSL4 | - | hsa-miR-3666 |  | ACSL4 | - | hsa-miR-4524b-5p |
| ACSL4 | - | hsa-miR-19a-3p |  | ACSL4 | - | hsa-miR-15a-5p |
| ACSL4 | - | hsa-miR-19b-3p |  | ACSL4 | - | hsa-miR-16-5p |
| ACSL4 | - | hsa-miR-129-5p |  | ACSL4 | - | hsa-miR-15b-5p |
| ACSL4 | - | hsa-miR-204-5p |  | ACSL4 | - | hsa-miR-195-5p |
| ACSL4 | - | hsa-miR-211-5p |  | ACSL4 | - | hsa-miR-944 |
| ACSL4 | - | hsa-miR-9-3p |  | ACSL4 | - | hsa-miR-150-5p |
| ACSL4 | - | hsa-miR-582-5p |  | ACSL4 | - | hsa-miR-524-5p |
| ACSL4 | - | hsa-miR-374a-5p |  | ACSL4 | - | hsa-miR-520d-5p |
| ACSL4 | - | hsa-miR-17-5p |  | ACSL4 | - | hsa-miR-449b-5p |
| ACSL4 | - | hsa-miR-20a-5p |  | CHAC1 | - | hsa-miR-15a-5p |
| ACSL4 | - | hsa-miR-93-5p |  | CHAC1 | - | hsa-miR-16-5p |
| ACSL4 | - | hsa-miR-106a-5p |  | CHAC1 | - | hsa-miR-15b-5p |
| ACSL4 | - | hsa-miR-7-5p |  | CHAC1 | - | hsa-miR-195-5p |
| ACSL4 | - | hsa-miR-106b-5p |  | CHAC1 | - | hsa-miR-6838-5p |
| ACSL4 | - | hsa-miR-20b-5p |  | CHAC1 | - | hsa-miR-424-5p |
| ACSL4 | - | hsa-miR-526b-3p |  | CHAC1 | - | hsa-miR-497-5p |
| ACSL4 | - | hsa-miR-519d-3p |  | CHAC1 | - | hsa-miR-1297 |
| ACSL4 | - | hsa-miR-374c-5p |  | CHAC1 | - | hsa-miR-26a-5p |
| ACSL4 | - | hsa-miR-374b-5p |  | CHAC1 | - | hsa-miR-26b-5p |
| ACSL4 | - | hsa-miR-186-5p |  | ENPP2 | - | hsa-miR-670-3p |
| ACSL4 | - | hsa-miR-519c-3p |  | ENPP2 | - | hsa-miR-101-3p |
| ACSL4 | - | hsa-miR-519b-3p |  | ENPP2 | - | hsa-miR-29a-3p |
| ACSL4 | - | hsa-miR-519a-3p |  | ENPP2 | - | hsa-miR-29b-3p |
| ACSL4 | - | hsa-miR-655-3p |  | ENPP2 | - | hsa-miR-29c-3p |
| ACSL4 | - | hsa-miR-3164 |  | FAT1 | - | hsa-miR-641 |
| ACSL4 | - | hsa-miR-4524a-5p |  | FAT1 | - | hsa-miR-3617-5p |
| ACSL4 | - | hsa-miR-34a-5p |  | FAT1 | - | hsa-miR-105-5p |
| ACSL4 | - | hsa-miR-181a-5p |  | SQLE | - | hsa-miR-579-3p |
| ACSL4 | - | hsa-miR-181b-5p |  | SQLE | - | hsa-miR-664b-3p |
| ACSL4 | - | hsa-miR-181c-5p |  | SQLE | - | hsa-miR-133a-3p |
| ACSL4 | - | hsa-miR-181d-5p |  | SQLE | - | hsa-miR-133b |

“mRNA”and“miRNA”represent node；“-”represent edge.
