# Supplementary material for: Use of a ferroptosis-related gene signature to construct diagnostic and prognostic models for assessing immune infiltration in metabolic dysfunction-associated fatty liver disease
Source: Front Cell Dev Biol. 2023 Oct 19;11:1199846. doi: 10.3389/fcell.2023.1199846 (PMC10622674; doi:10.3389/fcell.2023.1199846)
Supplement: Supplementary file 7 [file Table3.docx]

### Table S3. KEGG enrichment analysis results of Ferroptosis-related differentially expressed genes.

| Ontology | ID | Description | GeneRatio | BgRatio | pvalue | p.adjust | qvalue |
| --- | --- | --- | --- | --- | --- | --- | --- |
| KEGG | hsa01212 | Fatty acid metabolism | 4/10 | 57/8076 | 4.54e-07 | 1.18e-05 | 8.60e-06 |
| KEGG | hsa03320 | PPAR signaling pathway | 4/10 | 78/8076 | 1.62e-06 | 2.10e-05 | 1.53e-05 |
| KEGG | hsa00061 | Fatty acid biosynthesis | 2/10 | 18/8076 | 2.09e-04 | 0.002 | 0.001 |
| KEGG | hsa01040 | Biosynthesis of unsaturated fatty acids | 2/10 | 27/8076 | 4.76e-04 | 0.003 | 0.002 |
| KEGG | hsa04152 | AMPK signaling pathway | 2/10 | 120/8076 | 0.009 | 0.047 | 0.035 |

KEGG：Kyoto Encyclopedia of Genes and Genomes.
