# Supplementary material for: Use of a ferroptosis-related gene signature to construct diagnostic and prognostic models for assessing immune infiltration in metabolic dysfunction-associated fatty liver disease
Source: Front Cell Dev Biol. 2023 Oct 19;11:1199846. doi: 10.3389/fcell.2023.1199846 (PMC10622674; doi:10.3389/fcell.2023.1199846)
Supplement: Supplementary file 9 [file Table4.docx]

### Table S4. GSEA analysis of Train dataset.

| Description | setSize | enrichmentScore | NES | pvalue | p.adjust |
| --- | --- | --- | --- | --- | --- |
| WP_TYROBP_CAUSAL_NETWORK | 53 | 0.81349 | 2.70540 | 0.00168 | 0.02478 |
| WP_CHOLESTEROL_METABOLISM_INCLUDES_BOTH_BLOCH_AND_KANDUTSCHRUSSELL_PATHWAYS | 40 | 0.78920 | 2.51794 | 0.00167 | 0.02478 |
| REACTOME_NEUTROPHIL_DEGRANULATION | 416 | 0.57421 | 2.48234 | 0.00126 | 0.02478 |
| WP_MICROGLIA_PATHOGEN_PHAGOCYTOSIS_PATHWAY | 35 | 0.80722 | 2.47071 | 0.00168 | 0.02478 |
| WP_STEROL_REGULATORY_ELEMENTBINDING_PROTEINS_SREBP_SIGNALLING | 66 | 0.68496 | 2.35902 | 0.00161 | 0.02478 |
| REACTOME_INTERLEUKIN_10_SIGNALING | 41 | 0.72002 | 2.30750 | 0.00166 | 0.02478 |
| REACTOME_ACTIVATION_OF_GENE_EXPRESSION_BY_SREBF_SREBP_ | 38 | 0.72363 | 2.27933 | 0.00166 | 0.02478 |
| REACTOME_CHOLESTEROL_BIOSYNTHESIS | 18 | 0.85135 | 2.27809 | 0.00185 | 0.02478 |
| KEGG_FC_GAMMA_R_MEDIATED_PHAGOCYTOSIS | 82 | 0.63473 | 2.27340 | 0.00159 | 0.02478 |
| PID_UPA_UPAR_PATHWAY | 41 | 0.70881 | 2.27159 | 0.00166 | 0.02478 |
| WP_TYPE_II_INTERFERON_SIGNALING_IFNG | 35 | 0.74128 | 2.26890 | 0.00168 | 0.02478 |
| PID_TCR_PATHWAY | 56 | 0.67193 | 2.25804 | 0.00165 | 0.02478 |
| REACTOME_INTERLEUKIN_2_FAMILY_SIGNALING | 44 | 0.69819 | 2.25601 | 0.00166 | 0.02478 |
| KEGG_ALLOGRAFT_REJECTION | 29 | 0.76812 | 2.24949 | 0.00172 | 0.02478 |
| WP_CHOLESTEROL_BIOSYNTHESIS_PATHWAY | 12 | 0.90616 | 2.20273 | 0.00190 | 0.02478 |

GSEA : Gene Set Enrichment Analysis.
