# Supplementary material for: Use of a ferroptosis-related gene signature to construct diagnostic and prognostic models for assessing immune infiltration in metabolic dysfunction-associated fatty liver disease
Source: Front Cell Dev Biol. 2023 Oct 19;11:1199846. doi: 10.3389/fcell.2023.1199846 (PMC10622674; doi:10.3389/fcell.2023.1199846)
Supplement: Supplementary file 10 [file Table6.docx]

### Table S6. mRNA-TF interaction network nodes.

| mRNA |  | TF |  | mRNA |  | TF |
| --- | --- | --- | --- | --- | --- | --- |
| ACSL4 | - | CREB1 |  | ENO3 | - | E2F1 |
| ACSL4 | - | E2F1 |  | ENO3 | - | E2F6 |
| ACSL4 | - | GATA1 |  | ENO3 | - | EBF1 |
| ACSL4 | - | SPIB |  | ENO3 | - | EHF |
| CHAC1 | - | ZEB1 |  | ENO3 | - | ERG |
| CHAC1 | - | CEBPA |  | ENO3 | - | ETS1 |
| CHAC1 | - | CEBPB |  | ENO3 | - | FOS |
| CHAC1 | - | CREB1 |  | ENO3 | - | GABPA |
| CHAC1 | - | CTCF |  | ENO3 | - | KLF4 |
| CHAC1 | - | E2F1 |  | ENO3 | - | KLF5 |
| CHAC1 | - | E2F8 |  | ENO3 | - | KLF9 |
| CHAC1 | - | EGR1 |  | ENO3 | - | MTA3 |
| CHAC1 | - | ELF1 |  | ENO3 | - | MYB |
| CHAC1 | - | ERG |  | ENO3 | - | NFIC |
| CHAC1 | - | FOXA1 |  | ENO3 | - | SPI1 |
| CHAC1 | - | GLI2 |  | ENO3 | - | SRF |
| CHAC1 | - | GMEB2 |  | ENO3 | - | TBP |
| CHAC1 | - | IRF1 |  | ENO3 | - | TEAD4 |
| CHAC1 | - | KLF5 |  | ENO3 | - | ZBTB7A |
| CHAC1 | - | KLF9 |  | ENPP2 | - | AR |
| CHAC1 | - | MYB |  | ENPP2 | - | FOXA1 |
| CHAC1 | - | MYOD1 |  | ENPP2 | - | FOXA2 |
| CHAC1 | - | NFYA |  | FAT1 | - | AR |
| CHAC1 | - | PAX5 |  | FAT1 | - | ATF2 |
| CHAC1 | - | SP1 |  | FAT1 | - | CEBPB |
| CHAC1 | - | TCF12 |  | FAT1 | - | CREB1 |
| CHAC1 | - | TFAP4 |  | FAT1 | - | E2F1 |
| ENO3 | - | ARNT |  | FAT1 | - | E2F7 |
| ENO3 | - | BCL6 |  | FAT1 | - | SPI1 |
| ENO3 | - | BHLHE40 |  | FAT1 | - | TCF21 |
| ENO3 | - | CEBPA |  | SQLE | - | CTCF |
| ENO3 | - | CEBPB |  | SQLE | - | E2F1 |
| ENO3 | - | CEBPD |  | SQLE | - | KLF5 |
| ENO3 | - | CTCF |  |  |  |  |

“mRNA”and“TF”represent node；“-”represent edge；TF：Transcription factors.
